# Establishment of bovine expanded potential stem cells

**DOI:** 10.1073/pnas.2018505118

**Published:** 2021-04-08

**Authors:** Lixia Zhao, Xuefei Gao, Yuxuan Zheng, Zixin Wang, Gaoping Zhao, Jie Ren, Jia Zhang, Jian Wu, Baojiang Wu, Yanglin Chen, Wei Sun, Yunxia Li, Jie Su, Yulin Ding, Yuan Gao, Moning Liu, Xiaochun Bai, Liangzhong Sun, Guifang Cao, Fuchou Tang, Siqin Bao, Pentao Liu, Xihe Li

**Affiliations:** ^a^The State Key Laboratory of Reproductive Regulation and Breeding of Grassland Livestock, Inner Mongolia University, 010070 Hohhot, China;; ^b^Research Center for Animal Genetic Resources of Mongolia Plateau, College of Life Sciences, Inner Mongolia University, 010070 Hohhot, China;; ^c^Inner Mongolia Saikexing Institute of Breeding and Reproductive Biotechnology in Domestic Animal, 011517 Hohhot, China;; ^d^Academy of Orthopedics, Guangdong Province, Department of Orthopedic Surgery, The Third Affiliated Hospital of Southern Medical University, 510630 Guangzhou, China;; ^e^Department of Physiology, School of Basic Medical Sciences, Southern Medical University, 510515 Guangzhou, China;; ^f^School of Biomedical Science, Stem Cell and Regenerative Consortium, Li Ka Shing Faculty of Medicine, The University of Hong Kong, 999077 Hong Kong;; ^g^Beijing Advanced Innovation Center for Genomics, College of Life Sciences, Peking University, 100871 Beijing, China;; ^h^College of Veterinary Medicine, Key Laboratory of Basic Veterinary Medicine, Inner Mongolia Agricultural University, 010018 Hohhot, China;; ^i^College of Veterinary Medicine, Key Laboratory of Clinical Diagnosis and Treatment Technology in Animal Disease, Inner Mongolia Agricultural University, 010018 Hohhot, China;; ^j^Department of Cell Biology, School of Basic Medical Sciences, Southern Medical University, 510515 Guangzhou, China;; ^k^Department of Pediatrics, Nanfang Hospital, Southern Medical University, 510515 Guangzhou, China;; ^l^Biomedical Institute for Pioneering Investigation via Convergence, Ministry of Education Key Laboratory of Cell Proliferation and Differentiation, 100871 Beijing, China;; ^m^Peking-Tsinghua Center for Life Sciences, Academy for Advanced Interdisciplinary Studies, Peking University, 100871 Beijing, China;; ^n^Centre for Translational Stem Cell Biology, Building 17W, The Hong Kong Science and Technology Park, 999077 Hong Kong

**Keywords:** bovine, expanded potential stem cell, nuclear transfer

## Abstract

Embryonic stem cells (ESCs) and induced pluripotent stem cells have the potential to differentiate to all cell types of an adult individual and are useful for studying development and for translational research. However, extrapolation of mouse and human ESC knowledge to deriving stable ESC lines of domestic ungulates and large livestock species has been challenging. In contrast to ESCs that are usually established from the blastocyst, mouse expanded potential stem cells (EPSCs) are derived from four-cell and eight-cell embryos. We have recently used the EPSC approach and established stem cells from porcine and human preimplantation embryos. EPSCs are molecularly similar across species and have broader developmental potential to generate embryonic and extraembryonic cell lineages. We further explore the EPSC technology for mammalian species refractory to the standard ESC approaches and report here the successful establishment of bovine EPSCs (bEPSCs) from preimplantation embryos of both wild-type and somatic cell nuclear transfer. bEPSCs express high levels of pluripotency genes, propagate robustly in feeder-free culture, and are genetically stable in long-term culture. bEPSCs have enriched transcriptomic features of early preimplantation embryos and differentiate in vitro to cells of the three somatic germ layers and, in chimeras, contribute to both the embryonic (fetal) and extraembryonic cell lineages. Importantly, precise gene editing is efficiently achieved in bEPSCs, and genetically modified bEPSCs can be used as donors in somatic cell nuclear transfer. bEPSCs therefore hold the potential to substantially advance biotechnology and agriculture.

## Significance

Bovine embryonic stem cells and pluripotent stem cells hold the potential to substantially advance biotechnology and agriculture. We report the establishment of bovine expanded potential stem cells (bEPSCs) from preimplantation embryos of both wild-type and somatic cell nuclear transfer (SCNT). EPSCs have broader developmental potential to generate embryonic and extraembryonic cell lineages. bEPSCs express high levels of pluripotency genes, propagate robustly in single cell passaging, are genetically stable, and permit efficient precise gene editing. They differentiate in vitro and in chimeras to both the embryonic and extraembryonic cell lineages. Importantly, genetically modified bEPSCs can be used as donors in SCNT or cloning.

Embryonic stem cells (ESCs) of mouse, rat, human, and nonhuman primate species are established from the inner cell mass in the blastocyst (1–5). A significant advance in mouse ESC derivation is to use small molecule inhibitors, specifically the ones targeting Mek1/2 (PD0325901) and GSK3 (Chir99021), in the 2i/LIF naïve ESC culture condition (6). However, extrapolating the 2i/LIF condition to deriving ESCs of large animals has proven challenging. We and others recently reported establishment of mouse, human, and porcine expanded potential stem cells (EPSCs) (7–10). We posited that deriving stem cells from earlier preimplantation embryos, for example, mouse four-cell or eight-cell cleavage embryos, might help overcome the substantial species differences reported in the blastocyst-stage embryos (11–16). Following this line of reasoning, EPSCs were established by inhibiting molecules and pathways operating in preblastocyst embryos (7, 9, 10). EPSCs are molecularly and functionally similar across species. They possess robust self-renewal capacity in long-term culture, allow efficient genome editing, and generate both embryonic and extraembryonic cell lineages in vitro and in chimeras in terms of mouse and porcine EPSCs (7–9). Mouse EPSCs were reported to self-assemble into blastocyst-like structures (17).

Cattle, or cows, are domesticated bovine farm animals and the most common type of large domesticated ungulates. Bovine ESCs would be expected to substantially facilitate genome editing, to accelerate molecular breeding schemes for economic traits, and to provide a platform for investigating the bovine preimplantation development with potential applications in improving cloning. Intensive efforts have been made to derive bovine ESCs or to reprogram bovine somatic cells to induced plurpotent stem cells (iPSCs) (18–38). These cells, in general, however, lack the standard pluripotent stem cell criteria: poor derivation efficiencies; inability of maintaining pluripotency in long-term culture; limited developmental potential in the in vitro and in vivo assays such as chimera generation. The reported bovine iPSC lines often have leaky expression of the reprogramming genetic factors (28–38). Recently, bovine-primed ESCs were reported (39), which represents a major advance. These cells had the typical mouse- and human-primed ESC properties but morphologically did not form distinct cell colonies, unlike ESCs of other species, and no chimera was generated (39). In this report, we successfully established and characterized bovine EPSCs (bEPSCs). The availability of bEPSCs, which are robustness in culture, permit efficient genome editing, possess expanded developmental potential, are expected to substantially advance bovine stem cell biology to considerably facilitate selecting for superior animals for farming and to open up opportunities for biotechnology.

## Results

### Identification of a Culture Condition that Maintains Bovine Pluripotency.

**Fig. 1.**
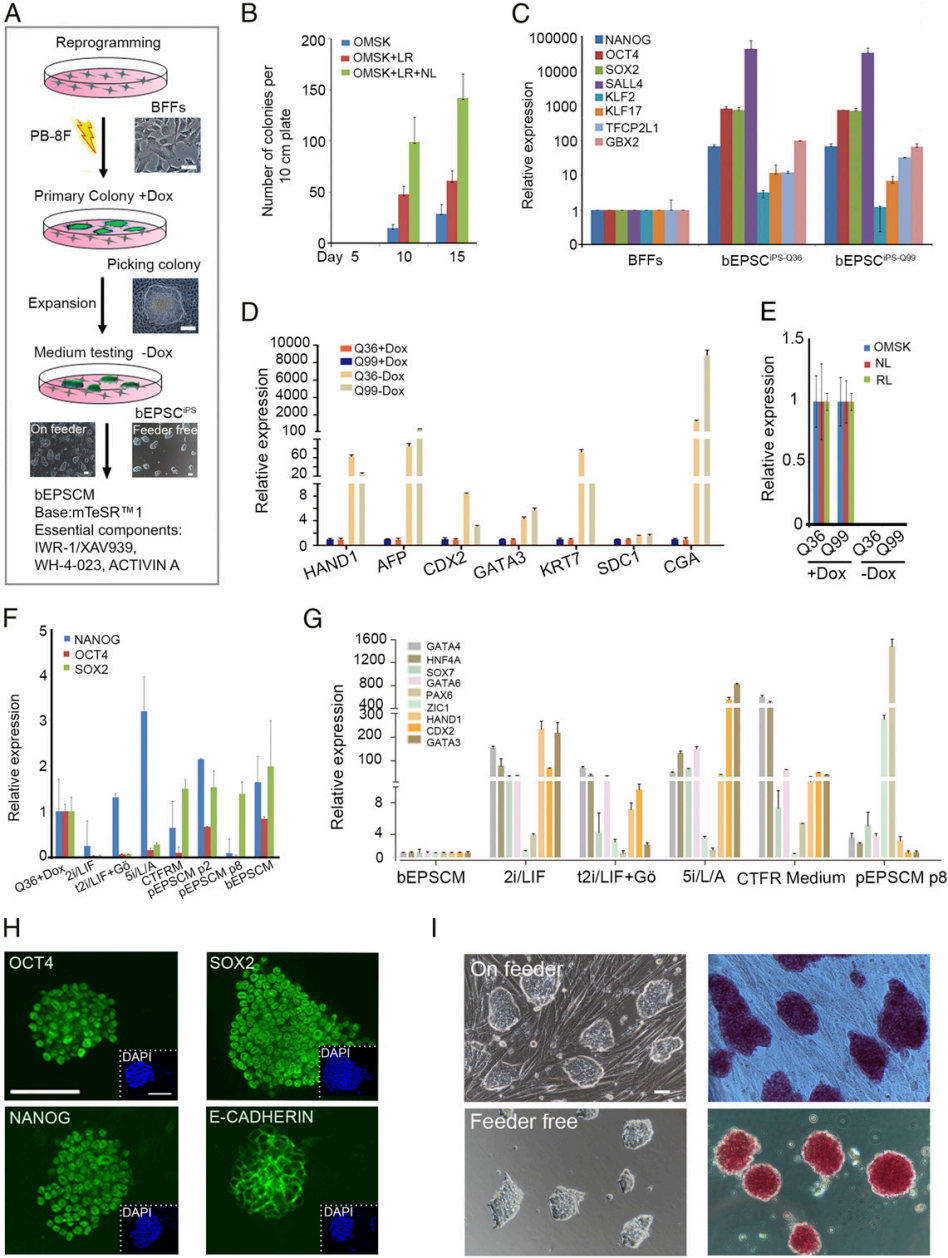
Reprogramming BFFs to Dox-inducible iPSCs for testing bovine stem cell culture condition. (*A*) Schematic illustration of reprogramming BFFs to iPSCs. PB-8F: bOMSK+ pN–hLIN + hRL. bOMSK (bovine OCT4, MYC, SOX2, and KLF4 cDNAs), pN–hLIN (porcine NANOG and human LIN28 cDNAs); hRL (human RARG and LRH1 cDNAs). BFFs: bovine fetal fibroblasts; Dox: doxycycline. (Scale bar, 50 μm.) (*B*) Coexpression of LIN28 (L), NANOG (N), LRH1, and RARG (LR) along with four Yamanaka factors substantially increased the reprogrammed colony numbers. (*C*) Relative expression of key endogenous pluripotency genes in two iPSC lines cultured in bEPSCM, bEPSC^iPS-Q36^, and bEPSC^iPS-Q99^. Data represent the mean ± SD, *n* = 3 independent experiments. (*D*) Expression of lineage genes in RT-qPCR of iPSC lines in the presence or absence of Dox in M15 medium. Q36: biPS-Q36 with Dox, Q99: biPS-Q99 with Dox. Data represent the mean ± SD, *n* = 3 independent experiments. (*E*) No detectable leaky expression of the exogenous reprogramming factors in iPSCs in RT-qPCR. (*F* and *G*) RT-qPCR analysis of pluripotency (*F*) and lineage genes (*G*) in bovine iPSCs under several culture conditions in the absence of Dox. These conditions include 2i/LIF, t2iL+Gӧ, and 5i/L/A on day 8; CTFR medium (passage 4); and pEPSCM (cells of passage 2 and passage 8 for analyzing pluripotency genes, and cells of passage 8 for analyzing lineage genes). Cells cultured in bEPSCM for passage 36 were used in the analysis. pEPSCM: porcine expanded potential stem cells medium, bEPSCM: bovine expanded potential stem cells medium, CTFRM: custom TeSR1 base medium supplemented with FGF2 and IWR1. Data represent the mean ± SD, *n* = 3 independent experiments. (*H*) Immunostaining of NANOG, OCT4, SOX2, and E-CADHERIN in bovine****bEPSC^iPS^. (Scale bar, 100 μm.) (*I*) The morphology and alkaline phosphatase (AP) staining of bEPSC^iPS-Q36^ on feeder cells (*Upper*) or feeder free (*Lower*). (Scale bar, 50 μm.)

We wished to identify culture conditions under which bona fide bovine pluripotent stem cells could be derived and stably maintained. Due to the limited supply of bovine preimplantation embryos, we chose to initially establish bovine iPSC lines for testing culture conditions. We expressed Dox-inducible eight exogenous reprogramming factors, bOMSK (bovine *OCT4*, *CMYC*, *SOX2*, and *KLF4*), pNhL (porcine *NANOG* and human *LIN28*), and hRL (human *RARG* and *LRH1*) in bovine fetal fibroblasts (BFFs) of China Qinchuan bovine, delivered via *piggyBac* transposition (Fig. 1*A*). Dox induction reprogrammed ∼0.1% transfected BFFs to primary colonies, which were picked on day 15 through 20 (Fig. 1 *A* and *B*). The picked colonies were passaged in single-cell suspension in a serum containing medium (M15) in the presence of Dox. The passaged cells expressed high levels of the endogenous pluripotency genes, such as *NANOG*, *OCT4* (*POU5F1*), and *SOX2* (Fig. 1*C*), and could be maintained undifferentiated in Dox for at least 50 passages. They were thus named bovine iPSCs. Upon Dox removal, bovine iPSCs were differentiated in 8 d, concomitant with the increased expression of both embryonic and extraembryonic cell-lineage genes and with the loss of pluripotency gene expression (Fig. 1*D*). Importantly, these Dox-dependent bovine iPSCs did not appear to have detectable leaky expression of the exogenous reprogramming factors once Dox was removed from the culture (Fig. 1*E*). The pluripotency in these iPSCs thus depended on Dox-induced exogenous factor expression in the serum-containing medium. These bovine iPSCs thus provided a platform for identifying culture conditions that would be able to maintain endogenous pluripotency gene expression, independent of the Dox-induced exogenous factor expression. We tested culture conditions for mouse ESCs and human ESCs including 2i/LIF (6), t2iL+ Gӧ (40), 5i/L/A (41), the recently reported CTFR medium (mTeSR1 supplemented with FGF2 and IWR1) for bovine primed EPSCs (39), and porcine EPSC medium (pEPSCM) (9). Bovine iPSCs Q36 of passage 20 were cultured under these conditions for 8 d without Dox and were morphologically and transcriptionally examined. In 2i, t2iL+ Gӧ, and 5i/L/A, bovine iPSCs lost *OCT4* and *SOX2* expression (Fig.1*F*) and expressed high levels of both embryonic and extraembryonic cell-lineage genes (Fig. 1*G*). Concomitantly, the compact and domed EPSC colonies were now flat and appeared to be differentiated (*SI Appendix*, Fig. S1*A*). The CTFR medium for primed bovine ESCs, on the other hand, was able to maintain *NANOG* and *SOX2* expression, but *OCT4* expression was substantially decreased (Fig. 1*F*). Moreover, in CTFR, bovine iPSCs adopted a flat colony morphology (*SI Appendix*, Fig. S1*A*) and expressed high levels of the lineage marker genes (*GATA4*, *HNF4A*, *HAND1*, *GATA6*, *CDX2*, and *GATA3*) (Fig. 1*G*), indicating a primed state and partial differentiation. Porcine EPSC medium (pEPSCM) was able to maintain the iPSCs for up to 7 passages with an undifferentiated morphology and high expression of core pluripotency genes (Fig. 1*F*). However, under pEPSCM, bovine iPSCs were eventually differentiated in long-term culturing, accompanied with down-regulation of core pluripotency genes and expression of both embryonic and extraembryonic cell-lineage genes, with differentiated cell morphology (Fig. 1 *F* and *G* and *SI Appendix*, Fig. S1*A*), demanding further adjustment of pEPSCM for bovine stem cells. pEPSCM contains inhibitors targeting GSK3, SRC, and Tanykrases (9). We modified the pEPSCM and developed bEPSCM for bovine stem cells, which contains XAV939 (or IWR-1), CHIR99021, WH-4–023 or A419259, Vitamin C, ACTIVIN A, and LIF in mTeSR basal medium, whereas pEPSCM used N2B27 basal medium (9). Under bEPSC medium (bEPSCM), bovine iPSCs formed compact colonies and expressed high levels of endogenous pluripotency genes (Fig. 1 *A*, *F*–*H*, and *SI Appendix*, Fig. S1*B*). Importantly, bovine iPSCs did not have detectable leaky expression of exogenous reprogramming factors (*SI Appendix*, Fig. S1*C*), could be maintained for at least 63 passages without noticeable differentiation, and retained a normal karyotype (*SI Appendix*, Fig. S1*D*). At passage 63, 2n = 60. 46 out of 52 metaphases or 88%. In vitro and in teratomas, bovine iPSCs could differentiate into cells representative of both embryonic and extraembryonic cell-lineage genes (*SI Appendix*, Fig. S1 *E*–*G*) and were thus named as bEPSC^iPS^. One remarkable feature of bEPSC^iPS^ was that they remained undifferentiated in feeder-free condition for more than 30 passages and proliferated robustly (Fig. 1*I*). Similar to porcine EPSCs, bEPSCs were sensitive to Mek1/2 inhibition. When Mek1/2 inhibitor PD0325901 (1.0 µm) was added into bEPSCM, most bEPSCs died in 4 d, and the remaining cells lost *OCT4* expression in the *OCT4*-*mCherry* reporter bEPSCs (*SI Appendix*, Fig. S1*H*). In RT-qPCR analysis, the remaining cells also lost *OCT4* and *NANOG* expression and expressed high levels of *SOX2,* indicating differentiation (*SI Appendix*, Fig. S1*I*). These differentiated cells did not survive the next passaging in bEPSCM. Therefore, porcine and bovine EPSCs require higher levels of Mek1/2 signaling than that in the mouse and human.

### Establishment of bEPSC Lines from Preimplantation Embryos.

**Fig. 2.**
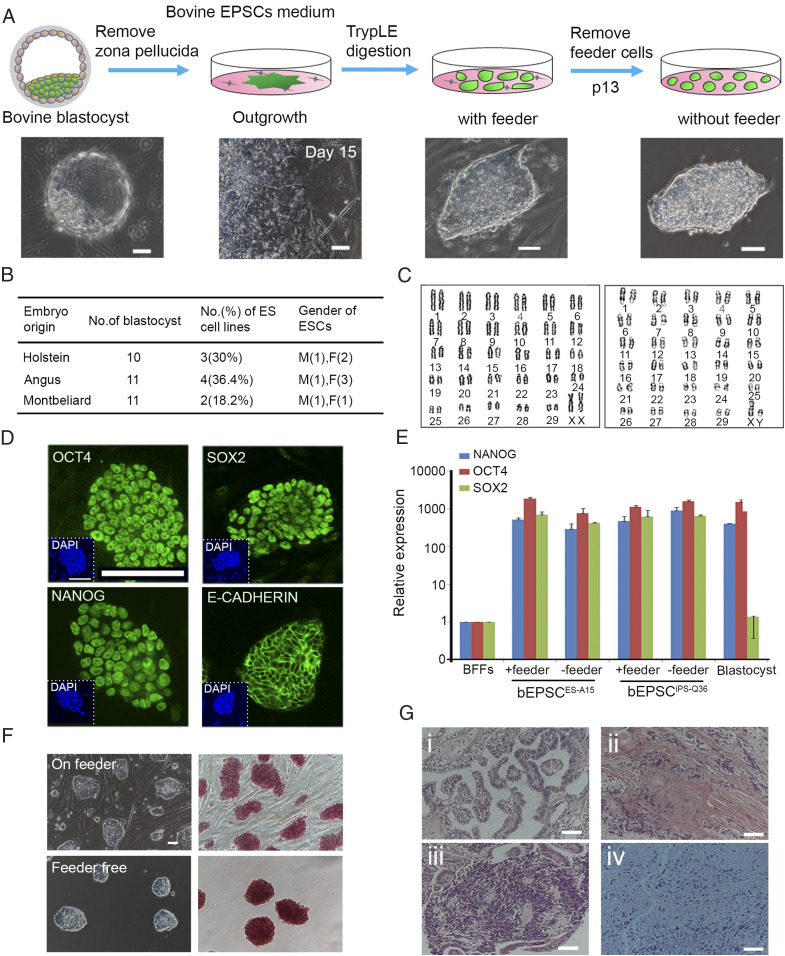
Establishment of bEPSCs from preimplantation embryos. (*A*) Schematic diagram of establishment of bEPSC^ES^ from bovine day 6 in vivo fertilization embryos. (Scale bar, 50 μm.) (*B*) bEPSC^ES^ lines of three breeds: Holstein, Angus, and Montbéliarde. (*C*) Karyotyping analysis of bEPSCs^ES-A6^ (female, passage 82) and bEPSCs^ES-A15^ (male, passage 76). (*D*) Immunostaining of NANOG, OCT4, SOX2, and E-CADHERIN in bEPSCs ^ES-A15^ of passage 38. (Scale bar, 100 μm.) (*E*) Relative expression of core pluripotency genes OCT4, NANOG and SOX2 in two bEPSC lines (bEPSCs^ES-A15^ on passage 32 and bEPSC^iPS-Q36^ on passage 36) on feeders or feeder-free. The relative expressions above were normalized to control and housekeeping gene. Data represent the mean ± SD, *n* = 3 independent experiments. (*F*) Morphology and AP staining of bEPSC^ES-A15^ on feeder cells (*Upper*, passage 36) or feeder free (*Lower*, passage 30). (Scale bar, 50 μm.) (*G*) Teratoma derived from bEPSC^ES-A15^ (passage 40). (*H* and *E*) Analysis revealed the presence of glandular epithelium (endoderm, i), muscle (mesoderm, ii), cartilage (mesoderm, iii), and mature neural tissue (glia and neurons, ectoderm, iv). (Scale bar, 50 μm.)

We next investigated deriving EPSC lines from bovine preimplantation embryos (Fig. 2*A*). From 32 early blastocysts (5 to 6 d postcoitum [dpc]) of Holstein, Angus, and Montbeliarde bovine, nine cell lines (bEPSC^ES^, 3 male and 6 female) were established (Fig. 2*B*). bEPSC^ES^ had high nuclear/cytoplasmic ratios and formed compact domed colonies with smooth colony edges (Fig. 2*A*). They proliferated robustly and were routinely passaged in 2 to 3 d (1:4 passaging ratio) without the need of the Rho Associated Coiled-Coil Containing Protein Kinase (ROCK) inhibitor Y-27632, and could be maintained in long-term cultures (>82 passages). Cryopreserved bEPSCs could be readily recovered. They were genetically stable and retained a normal karyotype (Fig. 2*C*) (2n = 60, 38/50, 76%, passage 82 for female bEPSC^ES^, 2n = 60, 36/50, 72%, passage 76 for male bEPSC^ES^). bEPSC^ES^ expressed high levels of pluripotency genes but undetectable or minimal levels of lineage genes (Fig. 2 *D* and *E* and *SI Appendix*, Fig. S2 *A* and *B*). bEPSC^ES^ could also be maintained feeder free in long-term cultures (Fig. 2 *E* and *F*) and differentiated via embryoid body formation into cells expressing genes representative of cell types of the three germ layers and trophoblast-like cells (PL-1^+^) (*SI Appendix*, Fig. S2 *C* and *D*). In vivo, bEPSC^ES^ formed mature teratomas that contained cell types of the somatic germ layers (Fig. 2*G*). After transient expression of the *SOX17* transgene, both bEPSCs^iPS^ and bEPSCs^ES^ could generate cells in the Embryoid Bodies (EBs) expressing genes highly enriched in primordial germ cells such as *OCT4*, *NANOG*, *SOX17*, *TFAP2C*, *NANOS3*, and *BLIMP1* (*SI Appendix*, Fig. S2 *E* and *F*), similar to porcine and human EPSCs (9).

We determined the colony formation capability from individual bEPSCs and compared to the primed ESCs. Single bEPSC^iPS-Q36^ and bEPSC^ES-A6^ were picked by microcapillary and seeded into individual 96-wells in bEPSCM. The colony formation efficiency was 47.9% for bEPSC^iPS^ and 40.9% for bEPSC^ES^, whereas much lower efficiencies (2.4% and 1.7%, respectively) were found for the same cells cultured in the primed bovine ESC medium, CTFR (*SI Appendix*, Fig. S2 *G* and *H*).

### Transcriptomic and Epigenetic Features of bEPSCs.

**Fig. 3.**
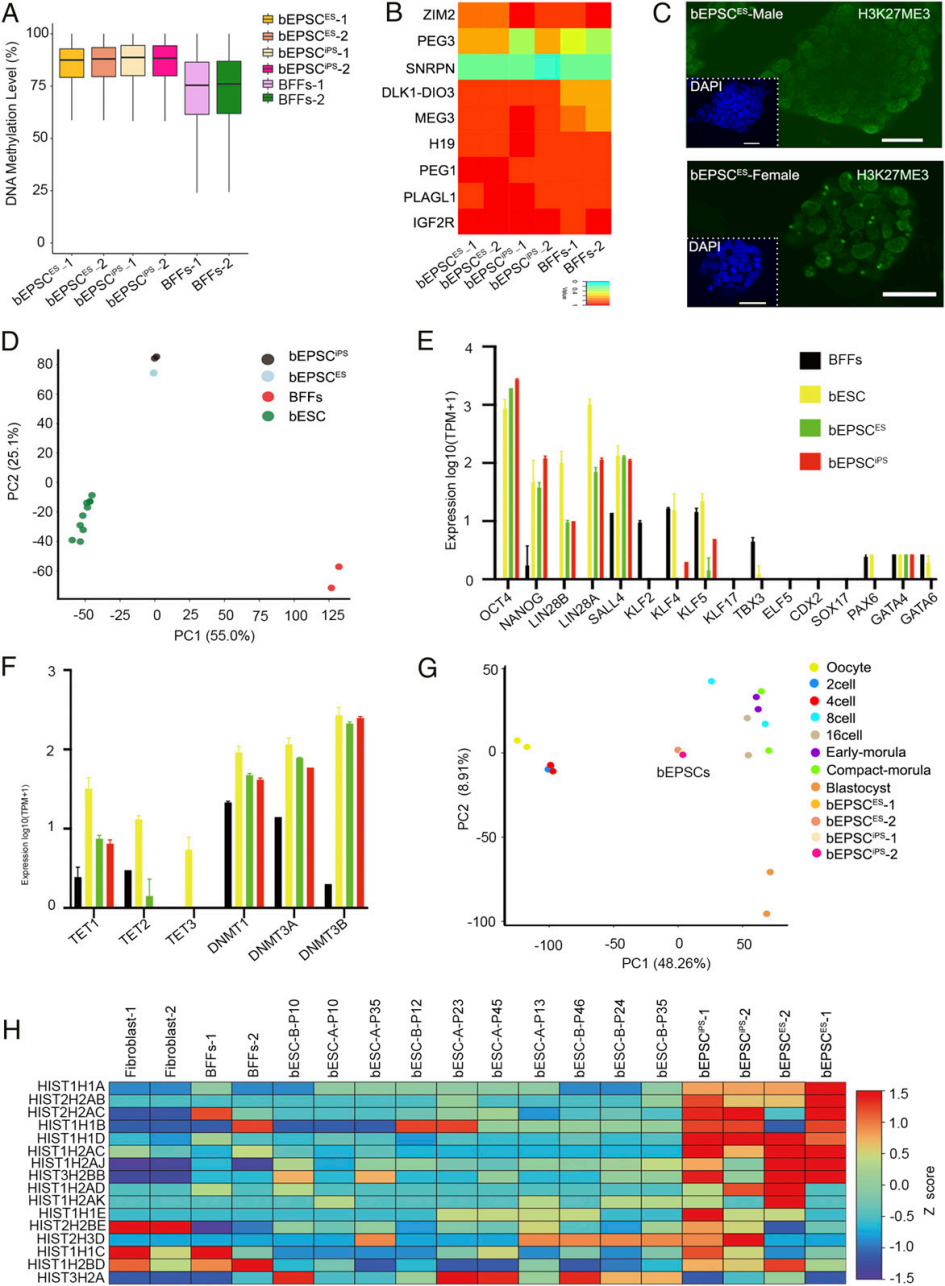
Transcriptomic and epigenetic features of bEPSCs. (*A*) DNA methylation levels in bEPSCs by whole-genome bisulfite sequencing analysis. Boxplots of the averaged DNA methylation levels (CpG sites) of 5 kb tiles in bEPSC^iPS-Q36^, bEPSC^ES-A6^, and BFFs. The bottom and top of the boxes indicate the first and third quartiles, respectively, and the lines inside the boxes indicate the medians of the data. (*B*) DNA methylation in DMR of bovine-imprinted genes, including *DLK1-DIO3* cluster, *H19* cluster, *IGF2R*, *MEG3*, *PEG3*, *PEG10*, *PLAGL1*, *SNRPPN*, and *ZIM2* in bEPSCs. (*C*) Immunostaining detection of H3K27me3 foci in female bEPSC^ES^. The male bEPSC^ES^ were the negative control. (Scale bar, 50 μm.) (*D*) PCA of global gene expression (RNA-seq) of bEPSCs, bovine-primed ESCs, and BFFs. (*E* and *F*) Expression of pluripotency genes, lineage genes, and DNA methylation genes in bEPSCs, pEPSCs, and BFFs. Bovine-primed ESCs, *n* = 10; BFFs, *n* = 2; bEPSCs^iPS-Q36^, *n* = 2; bEPSCs^ES-A6^, *n* = 2; *n* represents the number of biologically independent samples. (*G*) PCA of global gene expression (RNA-seq) of EPSCs and bovine preimplantation embryos (GSE59186) (60). Two replicates in each sample were used. (*H*) Expression levels of all annotated bovine histone genes in bovine-primed ESCs, EPSCs, and BFFs.

Unlike mouse and human naïve ESCs, human and porcine EPSCs have high levels of DNA methylation (9). Whole-genome bisulfite sequencing of bEPSC^ES^ and bEPSC^iPS^ revealed that DNA methylation levels in these cells were around 87%, with lower DNA methylation at the promoter regions, including bovine imprinting genes (Fig. 3 *A* and *B* and *SI Appendix*, Fig. S3 *A* and *B*). Proper genomic imprinting is required for normal mammalian development (42). Bovine preimplantation embryos have relatively low expression of some genomic imprinted genes (43–45). bEPSCs, in particular bEPSC^ES^, expressed low levels of imprinted genes *IGF2*, *IGF2R*, *H19*, *MEST*, *RTL1*, *PEG10*, *DLK1*, *DIO3*, *PEG3*, and *PLAGL1* (*SI Appendix*, Fig. S3*C*) (46–54). Compared to BFFs, bEPSCs exhibited comparable DNA methylation in the differential methylation regions at the genomic imprinting loci (Fig. 3*B* and *SI Appendix*, Fig. S3*B* and Table S1). X chromosome reactivation is an important indication in mouse and human naïve ESCs (55, 56). We sought to determine X reactivation state in female bEPSCs as this was not studied in the recently established bovine primed ESCs (39). We stained bEPSCs for H3K27me3 that is enriched on the inactivated X chromosome as a consequence of Xist-dependent recruitment of polycomb repressor complexes PRC2 (57, 58). We analyzed H3K27me3 staining domains or foci in cells of five colonies formed from cells of the two female lines, bEPSCs^ES-H4^ and bEPSCs^ES-A6^. In both cell lines, roughly 40% of the cells had the prominent perinuclear H3K27me3-positive foci (Fig. 3*C*). Interestingly, in female bovine preimplantation embryo development, X inactivation (XCI) starts as early as in morula, and its onset did not immediately lead to a global down-regulation of X-linked genes (59).

Global gene expression profiling of bEPSCs^ES^ and bEPSCs^iPS^ revealed that they were clustered together but distinct from primed bESCs and BFFs (Fig. 3*D*). Although expression of key pluripotency genes, such as *OCT4*, *SOX2*, *NANOG*, and *SALL4*, were similar between bEPSCs and primed bESCs (Fig. 3*E*), DNA methyltransferase genes, *DNMT1*, *DNMT3A*, and *DNMT3B*, were expressed at higher levels in bEPSCs, whereas *TET1*, *TET2*, and *TET3* were lower in bEPSCs (Fig. 3*F*), in line with high DNA methylation in bEPSCs. In comparison to bovine fibroblasts, bEPSCs had significantly higher expression of genes functioning in cell cycle and oxidative phosphorylation in Gene Set Enrichment Analysis (GSEA) (*SI Appendix*, Fig. S3*D*). Comparing to primed bovine ESCs, bEPSCs had higher gene expression relevant to MYC targets, DNA repair and oxidative phosphorylation in GSEA, whereas those genes in Notch signaling, Wnt signaling, and glycolysis were significantly overrepresented in primed bovine ESCs (*SI Appendix*, Fig. S3*E*). Transcriptomic analysis suggested that mouse EPSCs had enriched features of four- to eight-cell blastomeres (7), whereas human expanded potential stem cells (hEPSCs) were more similar to human eight-cell to morula-stage embryos than other developmental stages (9). Similarly, bEPSCs appeared to have enriched transcriptomic features of bovine eight-cell to morula stage embryos (60) in Principal Component Analysis (PCA) (Fig. 3*G*).

One unique feature of human EPSCs is high expression of certain histone genes (9). Indeed, bEPSCs, but not the primed bESCs, highly expressed many histone genes (Fig. 3*H*). Across the three species of human, pig, and bovine, EPSCs exhibited similar expression profiles of pluripotency genes and three germ-layer marker genes (*SI Appendix*, Fig. S3*F*), extraembryonic cell lineage genes (*SI Appendix*, Fig. S3*G*), and genes encoding enzymes for DNA methylation (*SI Appendix*, Fig. S3*H*).

### bEPSCs In Vivo Developmental Potential.

**Fig. 4.**
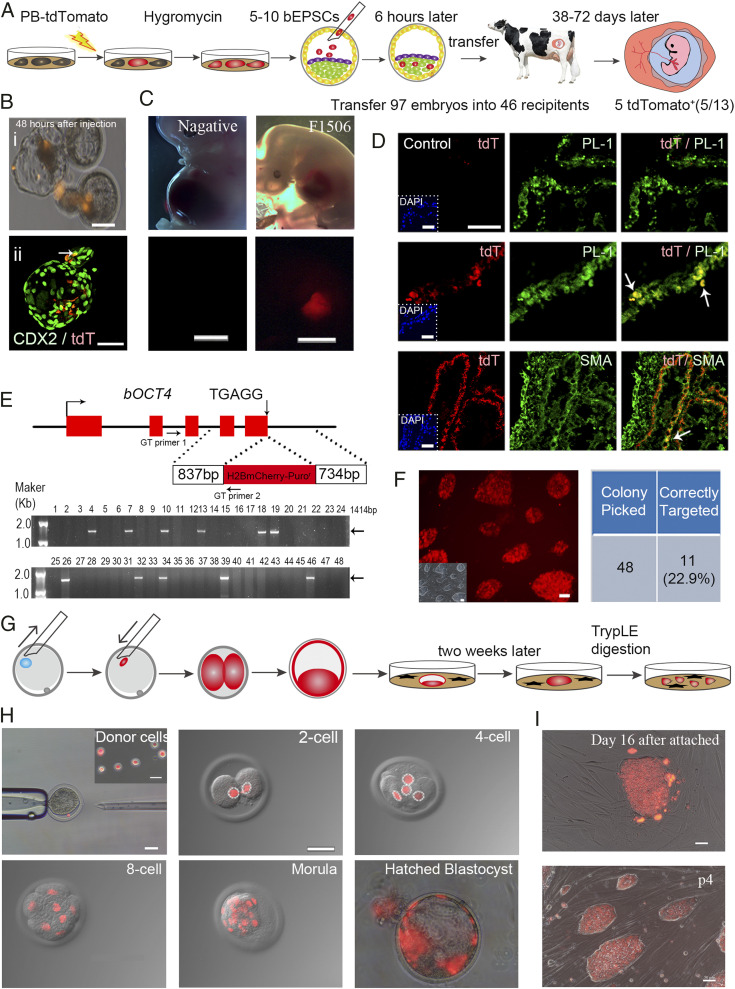
bEPSC’s developmental potential in chimeras. (*A*) Schematic diagram of chimera experiments using bEPSCs. (*B*) Contribution of bEPSCs in bovine preimplantation embryo development. The tdTomato^+^ donor bEPSCs^ES-A15^ (passage 20) were injected into bovine morula embryos, which developed into blastocysts in 48 h in vitro. Panel (i), Injected tdTomato^+^ bEPSCs^ES-A15^ in the blastocyst; Panel (ii), Several tdTomato^+^ cells (arrow) expressed trophectoderm factor CDX2. (Scale bar, 100 μm.) TdT, tdTomato. (*C*) Whole-mount fluorescence and bright field images of 40 d conceptuses from transferred preimplantation embryos. Chimera no. F1506 appeared to have tdTomato^+^ cells. (Scale bar, 0.5 cm.) (*D*) Detection of bEPSCs^ES-A15^ tdTomato^+^ descendants in the chorionic placenta (PL-1^+^; i) and in smooth muscles (SMA^+^; ii) in chimera F1506. The control embryos have no tdTomato^+^ cell injected. DAPI stains nuclei. (Scale bar, 50 μm.) TdT, tdTomato. (*E*) Genome editing in bEPSCs^ES-A15^. Knock-in of the T2A-H2B-mCherry cassette into the *OCT4* locus using the CRISPR/Cas9. The targeting vector with short homology arms from the *OCT4* locus flanking the T2A-H2B-mCherry and a Puromycin-resistance cassette was constructed and transfected into bEPSCs. The Puro-resistant transfectant colonies were picked 10 d after transfection. The correctly targeted colonies were identified in genomic DNA PCR. GT primers are for genotyping. (*F*) Bright field and fluorescence images of the correctly targeted bEPSCs colonies. Eleven out of forty-eight colonies examined were correctly targeted ones. (*G* and *H*) tdTomato^+^ bEPSC^ES-A15^ as the donor in SCNT. Injection of tdTomato^+^ bEPSC^ES-A15^ (passage 32) donor cells into the perivitelline space of oocytes was shown. (Scale bar, 50 μm.) (*I*) Derivation of secondary bEPSCs from SCNT (cloned) blastocysts. An outgrowth of day 16 from a SCNT blastocyst with bEPSCs as the donor cell (*Upper*) was picked for establishing the secondary EPSCs (*Lower*, Passage 4). (Scale bar, 50 μm.)

We next investigated the developmental capacity of bEPSCs in forming chimeras (Fig. 4*A*). bEPSC^ES-A15^ stably expressing tdTomato were generated by *piggyBac* transposition. We first tested bEPSCs in the mouse embryo development by injecting tdTomato^+^ bEPSCs into mouse eight-cell stage embryos and allowed the injected embryos to develop in vitro for 24 to 48 h into blastocysts. tdTomato^+^ cells were detected in both the trophectoderm and the inner cell mass of the mouse blastocysts (*SI Appendix*, Fig. S4*A*). The injected mouse morula embryos were also transferred to pseudopregnant female recipients for postimplantation development. From 62 transferred embryos, only 1 E6.5 chimera embryo was recovered where the donor origin tdTomato^+^ cells were primarily found in the extraembryonic ectoderm (ExE) region (*SI Appendix*, Fig. S4*B*). We also injected tdTomato^+^ bEPSCs to bovine morula embryos, which were allowed to further develop for 48 h to the blastocyst. tdTomato^+^ cells were detected in the inner cell mass (ICM) at 88.4% (46/52), 5.7% in the trophectoderm (3/52) (Fig. 4*B*). Some tdTomato^+^ cells (5 out of 32) in the trophectoderm (TE) were positive for CDX2 (Fig. 4*B*). To generate in vivo chimeras, we followed the blastocyst injection practice for producing mouse chimeras. We injected bovine early blastocysts (*n* = 97) with tdTomato^+^ bEPSCs and transferred them to pseudopregnant recipient cows (*n* = 46) (Fig. 4*A*). Thirteen recipients were found pregnant, and the embryos were harvested on day 38 (*n* = 4), day 40 (*n* = 7), and day 72 (*n* = 2) (Fig. 4*A*). Whole-mount fluorescence examination detected tdTomato^+^ cells in 5 of the 13 conceptuses (Fig. 4*C* and *SI Appendix*, Table S2). We further confirmed the presence of tdTomato^+^ cells by carefully dissecting and dissociating tdTomato^+^ tissues into single cells for fluorescence microscopy examination (Fig. 4*C* and *SI Appendix*, Fig. S4*C*). In two chimeras (No. F1607 and No. F1506, day 40), tdTomato^+^ cells were detected in both the placenta and embryonic tissues. We performed genomic DNA PCR to detect tdTomato DNA in various tissues of the five chimeras (*SI Appendix*, Fig. S4*D*) and genetically confirmed the presence of descendants of the donor tdTomato^+^ bEPSCs. To identify the descendants of donor bEPSCs in specific tissues, we performed immunofluorescence analysis to detect lineage marker expression in the tdTomato^+^ cells in chimeras, which revealed that tdTomato^+^ cells expressed markers of chorionic placenta (PL-1, GATA3, hCGβ, SDC1, and KRT7) and of embryonic cell lineages (SMA, β-TUBULIN III, SOX17, GATA6, and AFP) (Fig. 4*D* and *SI Appendix*, Fig. S4*E*). These results, together with the in vitro differentiation data (Fig. 1*D* and *SI Appendix*, Figs. S1 *E* and *F* and S2 *C* and *D*), indicate that bEPSCs have developmental potential to both the embryonic and extraembryonic cell lineages in chimeras, similar to porcine EPSCs (9).

### Precise Genome Editing in bEPSCs and SCNT Cloning.

Genome editing in bEPSCs would enable dissection of gene functions and advance biotechnology applications. Besides *piggyBac* transposition, we investigated precision genome editing in bEPSCs using the CRISPR system to knock an mCherry cassette into the bovine *OCT4* (*POU5F1*) locus (Fig. 4*E*). bEPSCs were transfected with a targeting vector where two short homology arms flanked a T2A-mCherry cassette. At the targeted allele, the mCherry coding sequence replaces the *OCT4* stop codon. Out of 48 genotyped colonies, 11 were correctly targeted for 22.9% targeting efficiency. The targeted bEPSCs were mCherry^+^ under fluorescence microscope (Fig. 4*F*). The efficient precise genome editing in bEPSCs enables sophisticated genome modifications in the bovine genome. We next explored using bEPSCs as donor cells in somatic cell nuclear transfer (SCNT). Nuclei from tdTomato^+^ bEPSCs^ES-A15^ were transferred into enucleated oocytes (*n* = 99), which were allowed to further develop to two-cell embryos (71.7%), eight-cell (41.4%), and the blastocyst (21.2%) (Table 1). These cloned embryo efficiencies were comparable to that of using fibroblasts as the control donors, indicating that bEPSCs could be used in SCNT for producing animals with sophisticated genetic modifications.

**Table 1.** bEPSCs as the donor in SCNT in comparison to bovine fibroblasts

Groups	No. of embryos reconstructed	No. of two-cell (%)	No. of eight-cell (%)	No. of blastocyst (%)
SCNT (fibroblast)	92	66 (71.7)	28 (42.4)	12 (17.1)
bEPSC^ES-A15^-NT	99	70 (71.7)	29 (41.4)	14 (21.2)

The SCNT embryos from bEPSCs provided an embryo source for testing deriving secondary EPSCs. Out of six SCNT blastocysts from bEPSCs^ES-A15^ as the donor, we derived one bEPSC line (Fig. 4 *G*–*I*). These secondary bEPSCs had typical EPSC morphology, retained a normal karyotype (2n = 60; 20 out of 25 metaphases at passage 12), and expressed pluripotency factors including SOX2, NANOG, and POU5F1 (*SI Appendix*, Fig. S4*F*), demonstrating the robustness of the bEPSC system.

## Discussion

Despite major advances in pluripotent stem cell research, establishing bovine ESCs comparable to the mouse and human counterparts is still challenging (18, 20, 26, 29–34, 36–38). In this study, we applied the EPSC technology to establish stem cell lines from bovine preimplantation embryos. We started reprogramming bovine somatic cells to Dox (exogeneous factors)-dependent iPSCs, which expressed high levels of endogenous pluripotency genes and thus allowed interrogating various culture conditions, including our published human and porcine EPSC culture conditions, for bovine stem cells. These experiments revealed that bovine stem cells necessitated a culture condition similar to porcine EPSC medium but demanded modifications and that, identical to porcine cells, bovine EPSCs required proper MEK1/2 activities as even low levels of the inhibitor PD-0325901 caused cell death and differentiation. A recent study reported bovine primed ESCs from the blastocyst (39), which marks a major advance in bovine stem cell research. In this study, we were able to reproduce the derivation of bovine primed ESCs. Compared to the primed ESCs, bEPSCs have several distinct properties that make bEPSCs the first bovine stem cells that****can substantially facilitate basic and applied research. First, bEPSCs have much higher single-cell subcloning efficiencies in the absence of the ROCK inhibitor, indicating a culture robustness. bEPSCs could even be maintained feeder-free in long-term culture. Second, the culture robustness of bEPSCs enables efficient precise genome editing, which would be challenging in the bovine primed ESCs. Importantly, genetically modified bEPSCs can serve as donor cells in SCNT. One remarkable feature of bEPSCs is the genetic stability. bEPSCs retained a normal karyotype and have high key pluripotency gene expression even after they were single cell–passaged for >82 passages. The secondary EPSCs, which are established from SCNT embryos of genetically modified bEPSCs as the donors, are still karyotypically normal. Third, bEPSCs are able to differentiate to various embryonic and extraembryonic cell lineages in chimeras, whereas primed ESCs are expected to have rare contribution in chimeras, based on mouse primed ESC data (61). Further research is needed to improve procedures including bEPSC culture, bEPSC injection, and embryo transfer for generating high quality live born chimeras. bEPSC’s genetic and epigenetic features, culture properties, efficient precise genome editing, and developmental potential provide a basis for applying bEPSCs in broad biotechnology and agriculture research areas. Importantly, the establishment of EPSCs of multiple mammalian species demonstrate that the EPSC technology could be applicable in additional mammals.

In summary, bEPSCs from preimplantation embryos and by reprogramming somatic cells are established and characterized for their molecular properties and developmental potential. These stem cells propagate robustly in long-term culture, permit precise genome editing, and generate both embryonic and extraembryonic cell lineages in vitro and in chimeras. bEPSCs represent bovine ESCs that are anticipated to have many applications in agriculture and biotechnology.

## Materials and Methods

### Culturing bEPSCs.

bEPSCs were maintained on BFFs feeder layers, or without feeder cells, and enzymatically passaged every 2 to 3 d by a brief PBS washing followed by treatment for 2 min with TrypLE Select (Gibco, 12563-029). The cells were dissociated and centrifuged (300 *g* × 5 min) in K10 medium. K10 includes DMEM F12 (Gibco), 10% KSR (Gibco), 1× penicillin-streptomycin, and 1× MEM nonessential amino acids (Gibco). After removing supernatant, the bEPSCs were resuspended and seeded in bEPSCM. bEPSCM is mTeSR1 (STEMCELL, 85850)-based media. bEPSC media (500 mL) was prepared as follows: 485 mL mTeSR1 (STEMCELL), 5.0 mL 100× penicillin-streptomycin (Gibco), 0.1 mM 2-mercaptoethanol (Gibco), and the small molecules and cytokines 1 μM CHIR99021 (GSK3i; Selleck Chemicals, S2924), 0.3 μM WH-4-023 (Selleck Chemicals, S7565), 5 μM XAV939 (Sigma, X3004) or 5 μM IWR-1 (Selleck Chemicals, S7086), 50 μg ⋅ mL^−1^ Vitamin C (Sigma, 49752-100G), 10 ng ⋅ mL^−1^ LIF (Millipore, LIF1010), and 20.0 ng ⋅ mL^−1^ Activin A (R&D, 338-AC).

### In Vivo Chimera Assay.

Six to twelve tdTomato^+^ bEPSCs^iPS-Q36^ were injected gently into the Institute of Cancer Research (ICR) mice eight-cell stage embryo using a piezo-assisted micromanipulator attached to an inverted microscope (Zeiss, Eppendorf); the protocol was performed as previously described. The injected embryos were cultured in KSOM (Millipore) and bEPSCM mixture medium (1:1) at 37 °C in a 5% CO_2_ atmosphere overnight and then transferred to the uteri of pseudopregnant ICR mice at 2.5 dpc. The embryos were isolated at embryonic stage E6.5 to check chimeric contribution. Also 5 to 10 bEPSCs^ES-A15^ (tdTomato^+^) were injected into bovine morulae and early blastocysts with the aid of a piezo-driven micromanipulator in synthetic oviductal fluid (SOF) medium and bEPSCM mixture medium (1:1). After injection, bovine embryos were cultured in the same medium at 38.5 °C in 5% CO_2_ and 5% O_2_ for 6 to 48 h. The injected morula embryos were cultured for 24 to 48 h to the blastocysts for in vitro study. In the blastocysts, on average 13.2 cells were tdTomato^+^ cells. The injected early blastocysts were for the evaluation of postimplantation chimerism. After a short time culture, they were transferred to the uteri of pseudopregnant bovine at 7 dpc. At day 23 to 30 after transplantation, pregnancy was diagnosed by ultrasonography and Rapid Visual Pregnancy Test Kit (IDEXX, 99-41369). The fetuses were isolated at embryonic stage day 38 to 72 to check chimeric contribution.

### CRISPR/Cas9-Mediated Genome Editing in bEPSCs.

To target an T2A-H2B–mCherry-EF1a-Puro cassette to the bovine *OCT4* locus, OCT4 5′ and 3′ homology arms were amplified by PCR from bEPSCs (837-bp 5′ arm, Chr23: 27,986,458–27,987,294; 734-bp 3′ arm, Chr23: 27,987,203–27,987,937), according to NCBI Reference Sequence NC_037350.1. The sequence 5′- GTG​CCT​GCT-​CAC​CCC​AGG​AAT​GG -3 was designed as the target of gRNA/Cas9.

### Production of Nuclear Transfer Embryos Reconstructed with bEPSCs^ES^.

The bEPSCs^ES^ within passages 15 through 25 were dispersed to a single-cell suspension by TrypLE select (Invitrogen) and recovered in bEPSCM. They were used as donor cells for nuclear transfer (NT). Single bEPSCs were individually transferred to the perivitelline space of the recipient cytoplasts. Successfully reconstructed embryos were kept in modified SOF (mSOF) (containing 5 mg/mL cytochalasin B) for 2 h until activation. All fused embryos were further activated in 5 mM ionomycin for 5 min, followed by exposure to 2 mM 6-dimethylaminopurine in SOF for 4 h. After the activation, NT embryos were washed and transferred into 500 μL of SOF media covered with mineral oil in a four-well plate, under an atmosphere of 5% CO_2_, 5% O_2_, 90% N_2_. The cleavage rates were determined 48 h after culturing, and the blastocyst rates were determined 7 d after culturing.

*SI Appendix*, *SI Materials and Methods* and Tables S1–S4 include further details of the study materials and methods.

## Supplementary Material

Supplementary File

## Data Availability

All study data are included in the article and/or *SI Appendix*.
