# Supplementary material for: Establishment of bovine expanded potential stem cells
Source: Proc Natl Acad Sci U S A. 2021 Apr 8;118(15):e2018505118. doi: 10.1073/pnas.2018505118 (PMC8053967; doi:10.1073/pnas.2018505118)
Supplement: Supplementary File [file pnas.2018505118.sapp.pdf]

PNAS
www.pnas.org

Supplementary Information for

Establishment of Bovine Expanded Potential Stem Cells

Lixia Zhao[a,b,c,1], Xuefei Gao[d,e,f,1], Yuxuan Zheng[g,1], Zixin Wang[c], Gaoping Zhao[c], Jie Ren[g], Jia Zhang[a,b], Jian Wu[f], Baojiang Wu[a,b,c], Yanglin Chen[a,b], Wei Sun[b,c], Yunxia Li[b,c], Jie Su[c,h], Yulin Ding[i], Yuan Gao[c], Moning Liu[h], Xiaochun Bai[d,j], Liangzhong Sun[k], Guifang Cao[h], Fuchou Tang[g,l,m], Siqin Bao[a,b], Pentao Liu[f,n,2,], and Xihe Li[a,b,c,2]

[a]The State Key Laboratory of Reproductive Regulation and Breeding of Grassland Livestock, Inner Mongolia University, 010070 Hohhot, China; [b]Research Center for Animal Genetic Resources of Mongolia Plateau, College of Life Sciences, Inner Mongolia University, 010070 Hohhot, China; [c]Inner Mongolia Saikexing Institute of Breeding and Reproductive Biotechnology in Domestic Animal, 011517 Hohhot, China; [d]Academy of Orthopedics, Guangdong Province, Department of Orthopedic Surgery, The Third Affiliated Hospital of Southern Medical University, 510630 Guangzhou, China; [e]Department of Physiology, School of Basic Medical Sciences, Southern Medical University, 510515 Guangzhou, China; [f]School of Biomedical Science, Stem Cell and Regenerative Consortium, Li Ka Shing Faculty of Medicine, The University of Hong Kong, 999077 Hong Kong; [g]Beijing Advanced Innovation Center for Genomics, College of Life Sciences, Peking University, 100871 Beijing, China; [h]College of Veterinary Medicine, Key Laboratory of Basic Veterinary Medicine, Inner Mongolia Agricultural University, 010018 Hohhot, China; [i]College of Veterinary Medicine, Key Laboratory of Clinical Diagnosis and Treatment Technology in Animal Disease, Inner Mongolia Agricultural University, 010018 Hohhot, China; [j]Department of Cell Biology, School of Basic Medical Sciences, Southern Medical University, 510515 Guangzhou, China; [k]Department of Pediatrics, Nanfang Hospital, Southern Medical University, 510515 Guangzhou, China; [l]Biomedical Institute for Pioneering Investigation via Convergence, Ministry of Education Key Laboratory of Cell Proliferation and Differentiation, 100871 Beijing, China; [m]Peking-Tsinghua Center for Life Sciences, Academy for Advanced Interdisciplinary Studies, Peking University, 100871 Beijing, China; [n]Centre for Translational Stem Cell Biology, The Hong Kong Science and Technology Park, 999077 Hong Kong

[2]To whom correspondence may be addressed. Email: pliu88@hku.hk or lixh@imu.edu.cn

This PDF file includes:

SI Materials and Methods
Figures S1 to S4
Tables S1 to S4
SI References

SI Materials and Methods

The Bovine iPSCs Reprogramming. China Qinchuan bovine fetal (day 45) fibroblasts were planted on gelatinized T75 culture flask and cultured in M10 medium. They were dissociated with TrypLE™ Select (Gibco, 12563-029) and harvested for electroporation at 80% confluence (～1.0 × 10^6 cells per experiment). M10 medium formulation was as follows: knockout DMEM (Gibco, 10829-018), 10% FBS (Gibco), 1×Penicillin-Streptomycin (Gibco) and 1×MEM Non-Essential Amino Acids (Gibco). The transfections were performed using an Amaxa Nucleofector machine (Lonza) according to the manufacturer's protocol (Basic Nucleofector® Kit for Primary Mammalian Fibroblasts, VPI-1001, program U-23), with 6.0 μg DNA (2.0 μg PB–TRE-bOMSK (bovine *OCT4*, *cMYC*, *SOX2* and *KLF4*), 1.0 μg PB–TRE–pNhL (porcine *NANOG* and human *LIN28*), 1.0 μg PB–TRE–hRL (human *RARG* and *LRH1*), 1.0 μg PB–EF1a–transposase and 1.0 μg PB–EF1a–rTTA)(1, 2). After transfection, 0.2-0.5 million BFFs were seeded on mitomycin-inactivated BFFs feeders in M15 supplemented with LIF (10 ng/ml, Millipore, LIF1001), Vitamin C (Sigma, 49752), 10ng/ml bFGF (R&D, 233-FB-025) and Dox (1.0 μg/mL, Clontech, 631311) in 10-cm dishes. M15: knockout DMEM (Gibco, 10829-018), 15% FBS (BI, 04-002-1A), 1×Penicillin-Streptomycin (Gibco, 11140-050), 1×GlutaMAX (Gibco, 35050-061), 1×MEM Non-Essential Amino Acids (Gibco) and 0.1 mM 2-mercaptoethanol (Sigma, M6250). The culture media was changed every other day, and the colonies were picked in M15 supplemented with Dox at day 15-20 and maintained in the same medium. The colonies with endogenous core pluripotent makers *OCT4*, *SOX2* and *NANOG* activated by RT-qPCR assay were selected for establishing transgene independent iPSCs lines in different culture condition with Dox removing.

Screening for the Bovine EPSC Culture Conditions. Dox-dependent bovine iPSCs, with endogenous core pluripotent makers *OCT4*, *SOX2* and *NANOG* activated were dissociated in TrypLE™ Select (Gibco), and seeded in 24-well BFFs feeder plates at a density of 1.2×10^4 cells per well. The cells were cultured in M15 medium supplemented with Dox (Clontech, 631311), Vitamin C (Sigma) and 10ng/ml bFGF (R&D, 233-FB-025), and then switched to candidate medium withdrawing Dox. The candidates were 2i/LIF, t2iL+Gö, 5i/L/A, CTFR-medium and pEPSCM (1, 3-6). bEPSCM were prepared as above. Small molecules and cytokines were supplemented as indicated at the following final concentrations: CHIR99021, 0.2 μM or 1 μM; PD0325901 (Selleck Chemicals, S1036), 1.0μM; WH-4-023 (Selleck Chemicals, S7565) or A-419259 (Tocris, 3914), 0.3 μM; PKC inhibitor Gö6983 (Selleck Chemicals, S2911), 5.0 μM; vitamin C, 50 μg ml^{-1}; SB590885 (BRAF inhibitor), 0.5 μM; IWR-1 and XAV939, 5.0 μM; Y27632 (Selleck Chemicals, S1049), 10 μM; LIF, 10 ng ml^{-1}; bFGF, 10.0-100 ng ml^{-1} and Activin A, 20.0 ng ml^{-1}. The medium was refreshed daily, and the surviving cells were passaged at day 5 with 10 μM Y27632. Knockout DMEM (Gibco, 10829-018), DMEMF12 (Gibco, 11320-033) and mTeSR™1 (STEMCELL, 85850) were used as basal medium for culture screening.

Culturing Bovine EPSCs. Bovine EPSCs were maintained on BFFs feeder layers, or without feeder cells, and enzymatically passaged every 2-3 days by a brief PBS washing followed by treatment for 2 minutes with TrypLE™ Select (Gibco, 12563-029). The cells were dissociated and centrifuged (300 g × 5 minutes) in K10 medium. K10: DMEMF12 (Gibco), 10% KSR (Gibco), 1×Penicillin-Streptomycin and 1×MEM Non-Essential Amino Acids (Gibco). After removing supernatant, the bovine EPSCs were re-suspended and seeded in bEPSCM. bEPSCM is mTeSR™1 (STEMCELL, 85850) based media. bEPSCM media (500 ml) was prepared as follows: 485 ml mTeSR™1 (STEMCELL), 5.0 ml 100×penicillin-streptomycin (Gibco), 0.1 mM 2-mercaptoethanol (Gibco), and the following small molecules and cytokines, 1 μM CHIR99021 (GSK3i; Selleck Chemicals, S2924), 0.3 μM WH-4-023 (Selleck Chemicals, S7565), 5 μM XAV939 (Sigma, X3004) or 5 μM IWR-1 (Selleck Chemicals, S7086), 50 μg ml^{-1} Vitamin C (Sigma, 49752-100G), 10ng ml^{-1} LIF (Millipore, LIF1010), 20.0 ng ml^{-1} Activin A (R&D, 338-AC). Our published porcine EPSC medium has: 0.2 μM CHIR99021, 0.3 μM WH-4-023, 2.5 μM XAV939 or 2.0 μM IWR-1, 65.0 μg ml^{-1} vitamin C, 10.0 ng ml^{-1} LIF, 20.0 ng ml^{-1} Activin and 0.3% FBS.

All cell cultures in this paper were performed under conditions of 38.5 °C and 5% CO_2 unless stated otherwise. bEPSCs were frozen once they are ~80% confluent using cryopreservation

medium, which contains 90% FBS and 10% (vol/vol) DMSO. The bEPSCs should be stored with the density of 1.0-2.0 × 10^5 bEPSCs per cryotube. Before cryorecovery, BFFs feeder plates should be prepared at least 1 day in advance at a density of 1.2 × 10^4 cells per well (24 well plate), and 1.0-2.0 × 10^5 bEPSCs should be seeded to one well of feeder plates with bEPSCM.

Bovine Superovulation, Insemination and Embryo Recovery. Holsteins (approx. 14-18 months of age, 300-400 kg bodyweight) served as embryo donors. Bovine donors were received a CIDR-B device (DEC international NZ l, New Zealand) on day 0, and 1 mg estradiol benzoate (Ningbo Second Hormone Factory, China) was injected simultaneously. Then 200 mg FSH (Folltropin-V, Canada) was injected from day 5 to 8, twice injections daily, and with 0.6 mg PG (Shanghai Institute of Planned Parenthood Research, China) injection (twice daily) on Day 7. Then CIDR-B devices were removed on Day 8. Ovulation was induced by injection of Gonadorelin (Sansheng Biological Technology, China) 0.2 mg after onset of estrus on Day 9, and were inseminated 12 and 24 hours after onset of estrus. Morulae or blastocysts were recovered on Days 16 or 17, graded and cryopreservation for bEPSC derivation.

***In vitro* Culture of Bovine Preimplantation Embryos and Derivation of Bovine EPSCs^{ES}.** Bovine *in vivo* derived morulae or blastocysts from day 5-7 were used for the establishment of bovine EPSC lines. Zonae pellucidae of blastocysts were removed with Tyrode's solution, Acid (Sigma), then blastocysts were washed twice in SOF medium which includes MEM essential amino acids (GIBCO) and MEM non-essential amino acids (GIBCO) and 10 mg BSA ml^{-1} (SIGMA). Bovine blastocysts were cultured on a monolayer of Mitomycin C treated BFFs cells in bEPSC medium, supplemented with 10 μM Y27632 (Selleck) for 5 days, until initial outgrowths could be observed. Subsequently, bEPSCM medium without ROCKi was used for further culture. Medium was changed every other day. 12-20 days after plating, outgrowths were dissociated and passaged using TrypLE Select (Gibco) and were reseeded in the presence of the Rho kinase (ROCK) inhibitor Y-27632 (10 μM) into newly prepared wells with BFFs feeder cells. Growth of colonies was evaluated daily and approximately three days later cells began to form well-defined domed colonies. And then the bEPSCs^{ES} were passaged every 2-3 days at a ratio of 1:4 as single cells by TrypLE Select without the Rho kinase (ROCK) inhibitor Y-27632.

Bovine EPSC Feeder Free Cultivation and Characterization. Bovine EPSCs^{iPS} and EPSCs^{ES} on feeder cells were switched to plates coated with 20 μg/ml fibronectin (Millipore, FC010) and maintained in bEPSCM. The bEPSCs were passaged every 2-3 days at a ratio of 1:4 with single cell suspension. The chemical defined bEPSC in different passages were collected for the expression of core pluripotent makers detection by RT-qPCR, AP staining and immunofluorescence staining.

Bovine Single EPS Cell Proliferation Ability Assay. Dissociation of bovine EPSCs and primed ES cells was performed in TrypLE™ Select for 2 min at 38.5 °C, then resuspended with bEPSCM and CTFRM (3), carefully dissociated in a small drop of medium using blunted micro-capillaries with an inner diameter large enough to accommodate approximately single cell. Dissociated cells were then individually plated into single wells of 96-well (Corning) plates with BFFs feeder cells. After continuous culturing for 5-20 days, single colony detection and assessment were carried out under a dissecting microscope (Leica), and AP staining was conducted for further verification the single cell source. More than one colony in one cell was excluded.

***In vitro* EB Formation Assay of Bovine EPSCs.** *In vitro* differentiation, bovine EPSCs were detached from culture dishes using TrypLE Select, resuspended in 10% FBS DMEM without LIF and bFGF, and then seeded into ultra-low cell attachment U-bottom 96-well (Corning, 7007). After 4 days in suspension culture, EBs were transferred to gelatin-coated dishes and cultured for another 3 days prior to linage genes RT-qPCR and 7 days prior to immunostaining.

***In vivo* Teratoma Assay of Bovine EPSCs.** Bovine EPSCs (bEPSCs$^{iPS\text{-}Q36}$ and bEPSCs$^{ES\text{-}A6}$) were re-suspended in PBS supplemented with 30% matrigel (Corning, 354230) and 10 μM Rock

inhibitor Y-27632. Bovine EPSCs (5 × 10^6) were injected subcutaneously into both dorsal flanks of 8-week-old male NSG mice (Beijing Biocytogen) (100 μl per injection). EPSCs formed visible teratomas within 8 and 10 weeks. When the size of the teratomas reached ～1.2 cm^2, they were dissected, fixed overnight in 10% phosphate-buffered formalin and embedded in paraffin before sectioning.

***In vivo* Chimera Assay.** 6-12 $tdTomato^+$ $bEPSCs^{iPS\text{-}Q36}$ were injected gently into the ICR mice 8-cell stage embryo using a piezo-assisted micromanipulator attached to an inverted microscope (Zeiss, Eppendorf), the protocol was performed as previously described (7)·. The injected embryos were cultured in KSOM (Millipore) and bEPSCM mixture medium (1:1) at 37°C in a 5% CO_2 atmosphere overnight and then transferred to the uteri of pseudopregnant ICR mice at 2.5 days post coitus (dpc). The embryos were isolated at embryonic stage E6.5 to check chimeric contribution. And also 5-10 $bEPSCs^{ES\text{-}A15}$ ($tdTomato^+$) were injected into bovine morulae and blastocysts with the aid of a piezo-driven micromanipulator in SOF medium and bEPSCM mixture medium (1:1). After injection, bovine embryos were cultured in the same medium at 38.5 °C in 5% CO_2 and 5% O_2 for 6-48 hours, part of blastocysts for the evaluation of chimerism, and the other embryos were transferred to the uteri of pseudopregnant bovine at 7 dpc. At day 23-30 after transplantation, pregnancy was diagnosed by ultrasonography and Rapid Visual Pregnancy Test Kit (IDEXX, 99-41369). The fetuses were isolated at embryonic stage day 38–72 to check chimeric contribution.

CRISPR/Cas9-Mediated Genome Editing in bEPSC Cells. To target an T2A-H2B–mCherry-EF1a-Puro cassette to the bovine *OCT4* locus, OCT4 5' and 3' homology arms were amplified by PCR from bEPSCs (837-bp 5' arm, Chr23: 27,986,458–27,987,294; 734-bp 3' arm, Chr23: 27,987,203–27,987,937), according to NCBI Reference Sequence: NC_037350.1. The sequence 5'- GTGCCTGCTCACCCCAGGAATGG -3 was designed as the target of gRNA/Cas9.

Genotyping of Bovine Chimera Embryos and Gene Editing bEPSCs. Genomic DNA of bovine fetuses and bEPSCs were extracted from bEPSCs as described above(1) and of placentas using DNeasy Blood ＆Tissue Kit (QIAGEN, 69504). Genomic DNA PCR of tdTomato was employed to detect the presences of donor cells, and knock-in gene PCR with the primers flanking the insertion site of the T2A-H2BmCherry cassette was done for targeted cells selection. Amplification of a region in the bovine GAPDH served as the genomic DNA quality and PCR control. All PCR primers are listed in Key Resource Table.

Differentiation of Bovine EPSCs to PGCLCs. For transcription factor mediated bovine PGCLC induction experiments, the *piggyBac* based PB-CAG-SOX17-GR expression construct was electroporated into the bovine $EPSCs^{ES\text{-}A15}$ and $EPSCs^{iPS\text{-}Q36}$ with transposase plasmids. The SOX17 protein was fused with GR (human glucocorticoid receptor ligand-binding domain). bEPSCs harbouring the plasmids were selected by adding 150 μg/ml hygromycin (Gibco, 10687010) for four days. This system allows inducing the nuclear translocation of *SOX17* by addition of 2 μg/ml dexamethasone (Dex) (Sigma, D2915). For the pre-induction, bEPSCs were detached from the BFFs feeder layer by 5% Dispase (Gibrco, 17105-041) without dissociation and seeded on gelatinised plates in M15 media supplemented with 10 μM ROCKi Y-27632, 20 μg/ml Activin A (R&D) and 2.0 μg/ml Dex (Sigma, D2915). After the 12 hours of induction and pre-differentiation, the cells were collected using TrypLE™ Select (Gibco) and plated to ultra-low attachment U-bottom 96-well plates (Corning, 7007) at a density of 5,000–6,000 cells/well in 100 μl PGCLC medium 3-4 days later, then the EBs were collected for analysis. PGCLC medium is composed of Advanced RPMI 1640 (GIBCO, 12633-12), 1% B27 Supplement (Gibco, 17504044), 1×glutamine penicillin-streptomycin (Thermo Fisher Scientific, 11140-050), 1× NEAA (Thermo Fisher Scientific, 10378-016), 0.1 mM 2-mercaptoethanol (Sigma, M6250) and the following cytokines: 500 ng/ml BMP2 (R&D, 355-BM-010), 10 ng/ml human LIF, 100 ng/ml SCF (R&D, 255-SC-010), 50 ng/ml EGF (R&D, 236-EG-200) and 10 μM ROCK inhibitor (Y-27632).

Production of Nuclear Transfer Embryos Reconstructed with $bEPSCs^{ES}$. The $bEPSCs^{ES}$ within passages 15-25 were dispersed to a single cell suspension by TrypLE select (Invitrogen)

and recovered in bEPSCM. They were used as donor cells for NT. The NT protocol was performed as previously described (8). Briefly, matured oocytes were denuded of cumulus cells, and oocytes with the first polar body were transferred into TCM199-Hepes medium containing 7.5 μg/ml cytochalasin B. Enucleation was performed with a 20-mm (internal diameter) glass pipette by aspirating the first polar body and approximately 5% of the adjacent surrounding cytoplasm. Single bEPSCs were individually transferred to the perivitelline space of the recipient cytoplasts. Cell fusion was performed using two direct current pulses of 1.0 kV/cm for 10 μs by an ECM 830 Electroporation System (BTX, San Diego, CA, USA) in 0.30 M mannitol, 0.05 mM $CaCl_2$, 0.1 mM $MgCl_2$, and 0.05% BSA. Successfully reconstructed embryos were kept in modified synthetic oviductal fluid (mSOF) (containing 5 mg/mL cytochalasin B) for 2 hours until activation. All fused embryos were further activated in 5-mM ionomycin for 5 minutes, followed by exposure to 2 mM 6-dimethylaminopurine in SOF for 4 hours. After the activation, NT embryos were washed and transferred into 500 μl of SOF media covered with mineral oil in 4-well plate, under an atmosphere of 5% CO_2, 5% O_2, 90% N_2. The cleavage rates were determined 48h after culturing, and the blastocyst rates were determined 7 days after culturing.

Quantitative Real-Time PCR Analysis. Total RNA was isolated using a RNeasy Mini Kit (Qiagen, 74104) for cultured cells or RNeasy Micro Kit (Qiagen, 74004) for PGCL cells. Complementary DNA (cDNA) was prepared using a GoScript™ Reverse Transcription System (Promega， A5001). RT-qPCR primers are listed in Key Resource Table. KAPA SYBR FAST Universal qPCR Kit (Roche-KAPA, KK4601) were used for RT-qPCR assays. All RT-qPCR reactions were performed on Veriti 96 cell Thermal Cycler (Applied Biosystems, USA). Gene expression was determined relative to GAPDH using the ΔΔCt method. Data are shown as the mean and SD.

Cryosectioning and Immunofluorescence Staining. Immunofluorescence staining of bEPSCs was performed as described previously (9). The slides were imaged with a confocal microscope FLUOVIEW FV1000 (Olympus). For immunofluorescence staining of cryo-sections of chimeric fetus, all fetus were half separated along vertical axis, one half part for genotyping, and the other half part was immersed in 4% PFA immediately after collection overnight at 4°C, then dehydration 1 hour in 5% sucrose, then 10% sucrose 1 hour, 20% sucrose 1 hour. Next, fetuses were embedded in mixture of Tissue-Tek O.C.T. compound (SAKURA, 4583) and 20% sucrose (Sigma) overnight at 4 °C, at last for liquid nitrogen quick freezing. The frozen OCT blocks were sectioned by CRYOSTAR NX50 (Thermo), at 10 μm each section. Sections were first permeabilized with 0.1% Triton and blocked with 5 % donkey serum plus 1% BSA followed by incubations with primary antibodies overnight at 4 °C. The primary antibodies were used as follows: OCT4 (Santa Cruz Biotechnology), SOX2 (Millipore), NANOG (Thermo Fisher Scientific), AFP (R&D Systems), SMA (R&D Systems), PL-1 (SantaCruz), KRT7 (SantaCruz), SOX17 (R&D Systems), CDX2 (BioGenex), GATA6 (R&D Systems), hCGß (Abcam), SDC1 (Abcam), GATA3 (R&D Systems), Beta-III Tubulin (R&D Systems) and E-CADHERIN (BD Biosciences). Fluorescence-conjugated secondary antibodies were used to incubate the slides at room temperature for 1 hour. The antibodies are listed in Table S3.

RNA-seq Library Preparation and Sequencing. Total RNA was extracted from cells using RNeasy Mini Kit (Qiagen) following the manufacturer's instructions. After quality control by the Fragment Analyzer (Advanced Analytical), 1-2 μg of RNA was used to isolate mRNA according to the NEBNext Poly (A) mRNA Magnetic Isolation Module. Then, RNA-seq libraries were constructed under the manufacturer's instructions of the NEBNext ultra RNA library prep kit for Illumina (NEB). The generated libraries were pooled and sequenced on Illumina HiSeq 2000 platforms with 150-bp paired-end mode (sequenced by Novogene).

Whole Genome Bisulfite Sequencing (WGBS) Library Construction and Sequencing. The WGBS libraries were prepared according to the standard protocol (10, 11). Shortly, genomic DNA was isolated from cells using the DNeasy Blood & Tissue Kit (Qiagen) following the manufacturer's protocol. Then, approximately 2 μg of genomic DNA spiked with 10ng (0.5%) unmethylated lambda DNA (Fermentas) was sheared into an average size of 200-300 bp using

Covaris S2 system and purified with DNA Clean & Concentrator™-5 Kit (Zymo research). The fragmented DNA was end-repaired, dA-tailed and ligated with cytosine-methylated adapters (NEB), followed by cleanup of adaptor-ligated DNA using AMPure XP beads. Subsequently, bisulfite conversion was performed with EZ-96 DNA Methylation-Direct™ MagPrep (Zymo research) using the manufacture's standard manual. After bisulfite conversion, the converted templates were PCR amplified followed by purification and quantified using Qubit ds DNA high sensitivity dye (Invitrogen). The final quality-insured libraries were sequenced on Illumina HiSeq2000 platforms with 150-bp paired-end mode (sequenced by Novogene).

Processing RNA-Seq Data. RNA-seq raw sequencing data were firstly removed reads with adapters and low-quality bases using the customer script. Clean reads were then aligned into the bovine genome bosTau9 (UCSC version) with software TopHat (version: 2.0.12) using default parameters (12). Uniquely mapped reads were then counted with software HTSeq (13), and gene expression levels were quantified with the RPKM (reads per kilobase million).

Processing Whole Genome Bisulfite Sequencing (WGBS) Data. WGBS raw sequencing data were firstly removed first 9-bp random primers, and reads with adapters and low-quality bases using software trim_galore (version: 0.1.3) with parameters '--quality 20 --stringency 3 --length 50 --clip_R1 9 --clip_R2 9 --paired --trim1 --phred33'. Clean reads were then firstly paired aligned into the bovine genome bosTau9 (UCSC version) using software Bismark (version: 0.7.6) with parameters '--fastq --non_directional --unmapped --phred33-quals' (14). To improve the percentage of aligned reads, unmapped reads in a paired-end mode were then re-aligned into the same genome in a single-end mode. Uniquely mapped reads were then removed PCR duplicated reads with software SAMtools (version: 0.1.18) (15). For avoiding the sequencing bias, only reads with 3× coverage was used in the downstream analysis.

Batch Correction in Cross-Species Comparisons. Batch effects in RNA-seq data were obvious among different species and different studies which can be detected in principal component analysis (PCA), and therefore, batch correction was essential in cross-species comparisons. We set source studies corresponding to RNA-seq data as batch labels, and then removed batch effects with function ComBat in R package sva (16). The corrected RNA-seq data were used to perform PCA with R function prcomp and unsupervised clustering with R function hclust.

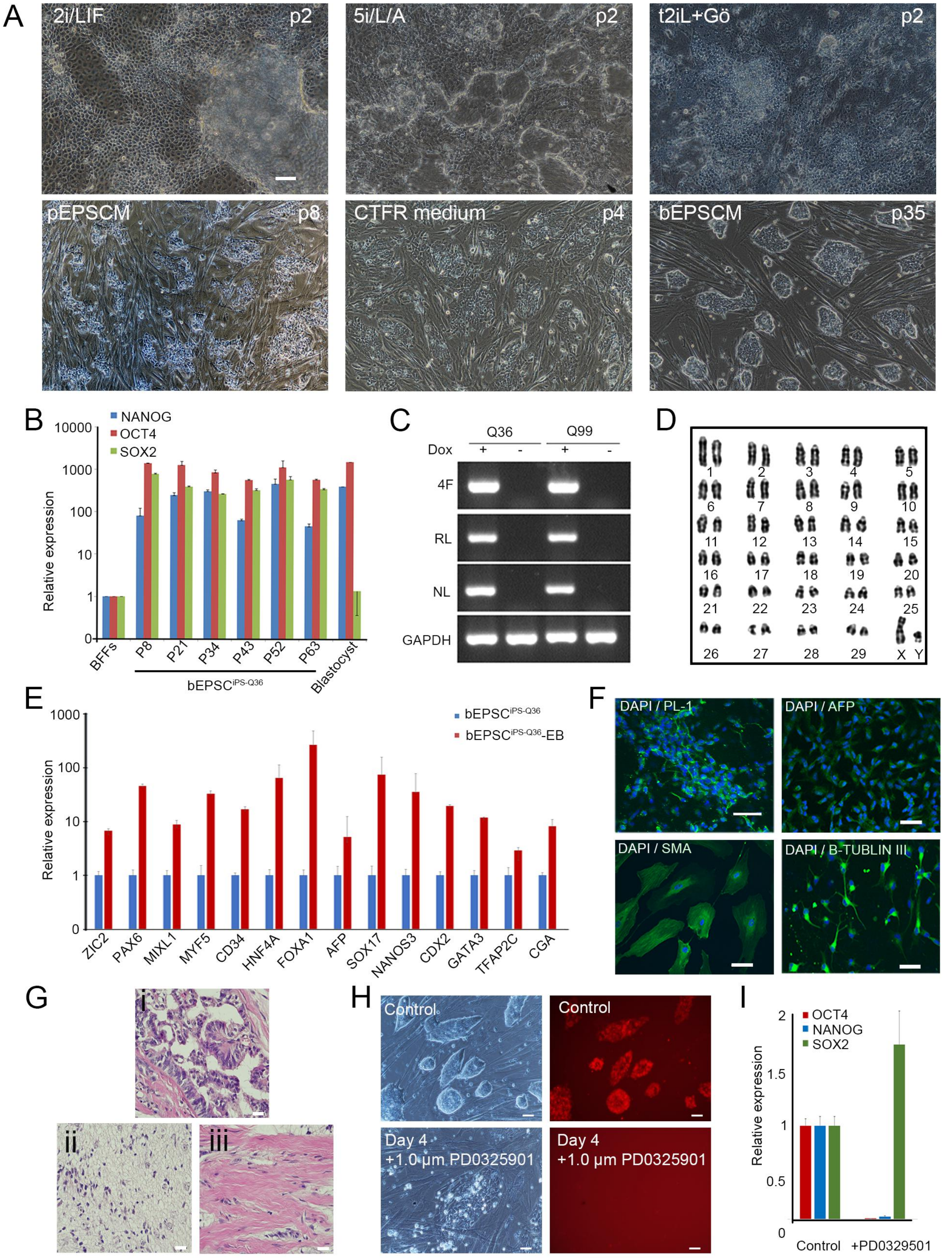

Fig. S1. Bovine iPSCs and EPSC^{iPS}. (*A*) Dox-dependent bovine iPSCs (iPSC-Q36) were cultured in several published media in the absence of Dox. These conditions include 2i/LIF, t2iL+Gö,

5i/L/A (cells cultured for day 8 in these conditions, passage 2); CTFR medium (cells cultured for 4 passages in this condition); pEPSCM (cells cultured for 8 passages in this condition); and bEPSCM (cells cultured for 35 passages in this condition). Cells cultured in porcine EPSCM were shown at passage 8 as they started to become differentiated. Bovine primed ESC medium CTFR is capable of keep bovine iPSCs undifferentiated in long-term culture. Scale bar, 100 μm. (*B*) Relative expression of the core pluripotency genes *OCT4*, *NANOG* and *SOX2* in bovine EPSCs $^{iPS\text{-}Q36}$ of various passages. Gene expression in the blastocyst was used as the control. The relative expression was normalized to BFFs and housekeeping gene *GAPDH*. Data are represented as mean ±SD, n = 3 independent experiments. (*C*) No detectable leaky expression of the exogenous reprogramming factors in 2 bovine EPSC^{iPS} lines by RT-qPCR when Dox was removed from the medium. (*D*) Karyotyping EPSCs$^{iPS\text{-}Q36}$ at passage 63 reveals that 46 out of 52 (88%) metaphase spreads had a normal karyotype. (*E*) Relative expression of lineage genes in bovine EPSC$^{iPS\text{-}Q36}$ embryoid body (EB) formation differentiation. The trophoblast cell markers include *CDX2*, *GATA3* and *CGA*. The relative expression was normalized to bEPSC$^{iPS\text{-}Q36}$ and housekeeping gene *GAPDH*. Data are represented as mean ±SD, n = 3 independent experiments. (*F*) Immunostaining of cells differentiated *in vitro* from bovine EPSC$^{iPS\text{-}Q36}$ for PL-1 (Placental lactogen I), AFP (Alpha-fetoprotein), SMA (Smooth muscle actin) and B-TUBLIN III. Scale bars, 50 μm. (*G*) Teratoma analysis of EPSC$^{iPS\text{-}Q36}$. H&E analysis detects the presence of glandular epithelium (i), neural tissue (ii), and muscle (iii). Scale bars, 50 μm. (*H*) Most bEPSCs were killed by 1.0 μM PD0325901 in four days, and the survived cells lost OCT4 expression indicated by mCherry$^-$ in *the OCT4-mCherry* reporter bEPSCs (see Fig. 4F). The control cells were cultured in bEPSCM. Scale bars, 50 μm. (*I*) RT-qPCR analysis of *OCT4*, *NANOG* and *SOX2* in the survival cells in bEPSCM plus 1.0 μM PD0325901 in four days indicates the differentiation to ectoderm cell lineages with high *SOX2* levels. The relative expression was normalized to bEPSCs and housekeeping gene *GAPDH*. Data are represented as mean ±SD, n = 3 independent experiments.

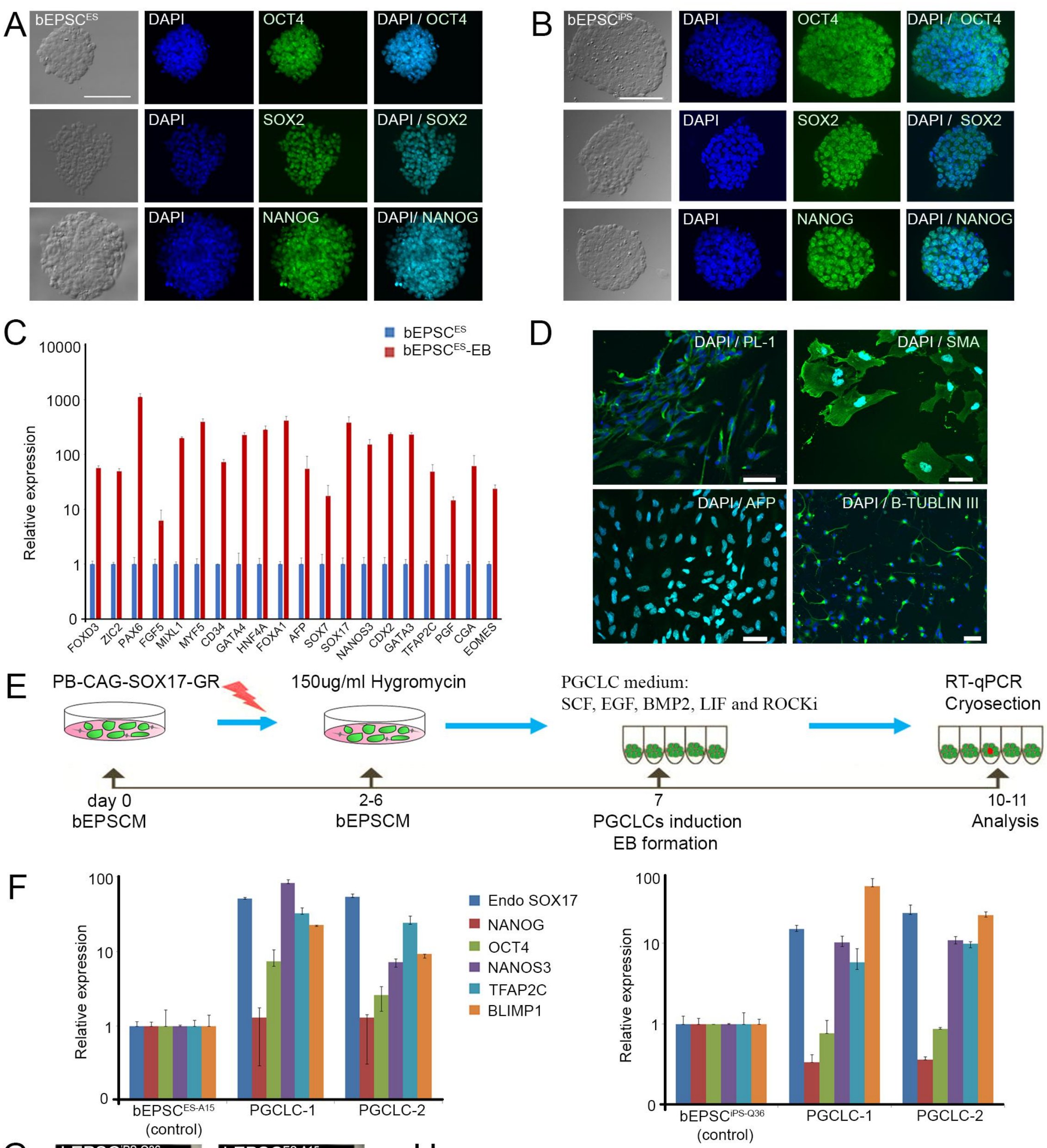

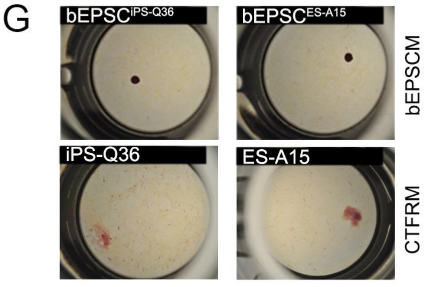

H

Culture medium	Type of cell	No. selected single cells	No. cell lines survived	The survival Rate
bEPSCM	bEPSC$^{iPS-Q36}$	288 (3x96)	138	47.9%
	bEPSC^{ES-A15}	288 (3x96)	118	40.9%
CTFRM	iPS-Q36	288 (3x96)	7	2.4%
	ES-A15	288 (3x96)	5	1.7%

Fig. S2. EPSCs derived from bovine preimplantation embryos - EPSC^{ES}. (*A-B*) Immunostaining of pluripotency factor in two feeder-free EPSC^{ES} lines bEPSCs^{ES-A15} (passage 17) and bEPSCs$^{iPS-Q36}$ (passage 26). Scale bars, 100 μm. (*C*) Relative expression of genes for the three germ layers

and the trophectoderm lineages in cells differentiated from $EPSC^{ES}$ via EB formation. The relative expression was normalized to $bEPSC^{ES}$ and housekeeping gene *GAPDH*. Data are represented as mean ±SD, n = 3 independent experiments. (*D*) Immunostaining of cells differentiated *in vitro* from bovine $EPSC^{ES\text{-}A15}$ for PL-1 (Placental lactogen I), AFP (Alpha-fetoprotein), SMA (Smooth muscle actin) and B-TUBLIN III. Scale bars, 100 μm. (*E*) Schematic illustration of bEPSC differentiation towards PGCLCs. After transiently expressing the *SOX17* transgene for 12 h, bEPSCs were cultured in PGCLC induction medium for 3-4 days for analysis. (*F*) Relative expression of PGC genes in day-3/4 EBs following PGC differentiation induction of $bEPSCs^{ES\text{-}A15}$ (left panels) and $bEPSCs^{iPS\text{-}Q36}$ (right panels). The relative expression was normalized to bEPSCs and housekeeping gene *GAPDH*. Data are represented as mean ±SD, n = 3 independent experiments. (*G*) AP staining of colonies derived from single EPSC cells of EPSCES-A15 under either bEPSCM or CTFRM on feeder cells. The colonies were AP stained at day 10 in bEPSCM (passage 36) or at day 20 in CTFRM (passage 18). (*H*) Summary of the colony numbers in the above experiments in G.

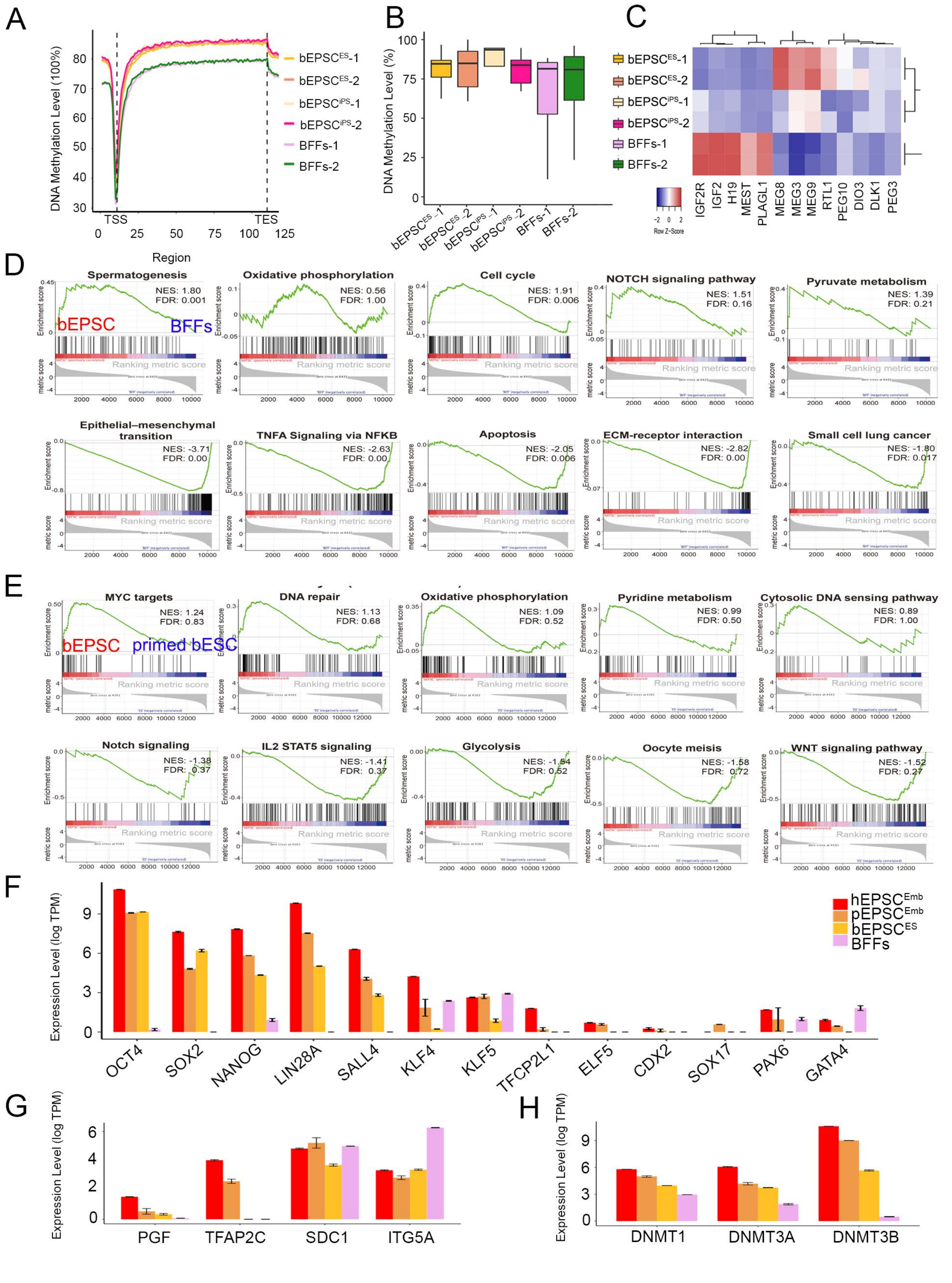

Fig. S3. Global epigenetic and transcriptome profile of bEPSCs. (*A*) Averaged DNA methylation levels along the gene bodies and 15 kilobases (kb) up- and downstream of the transcription start

sites (TSS) and transcription end site (TES), respectively, of all reference genes. (*B*) DNA methylation at the DMRs of several genomic imprinting loci. Table S1 has the detailed information about DMRs. (*C*) Expression of imprinted genes in bEPSCs and in fibroblasts. The cell lines are listed on the left. (*D*) Gene set enrichment analysis (GSEA) of bEPSCs and BFFs. Green line shows enrichment profile. Vertical black bars show where genes from a given gene set are located (hit). NES, normalized enrichment score; FDR, false discovery rate. (*E*) Gene set enrichment analysis (GSEA) of bEPSCs and primed bESCs. Green line shows enrichment profile. Vertical black bars show where genes from a given gene set are located (hit). NES, normalized enrichment score; FDR, false discovery rate. (*F-H*) Expression of pluripotency (*F*), lineage genes (*G*) and genes encoding enzymes for DNA methylation (*H*) in human EPSCs, porcine EPSCs, bEPSCs and BFFs.

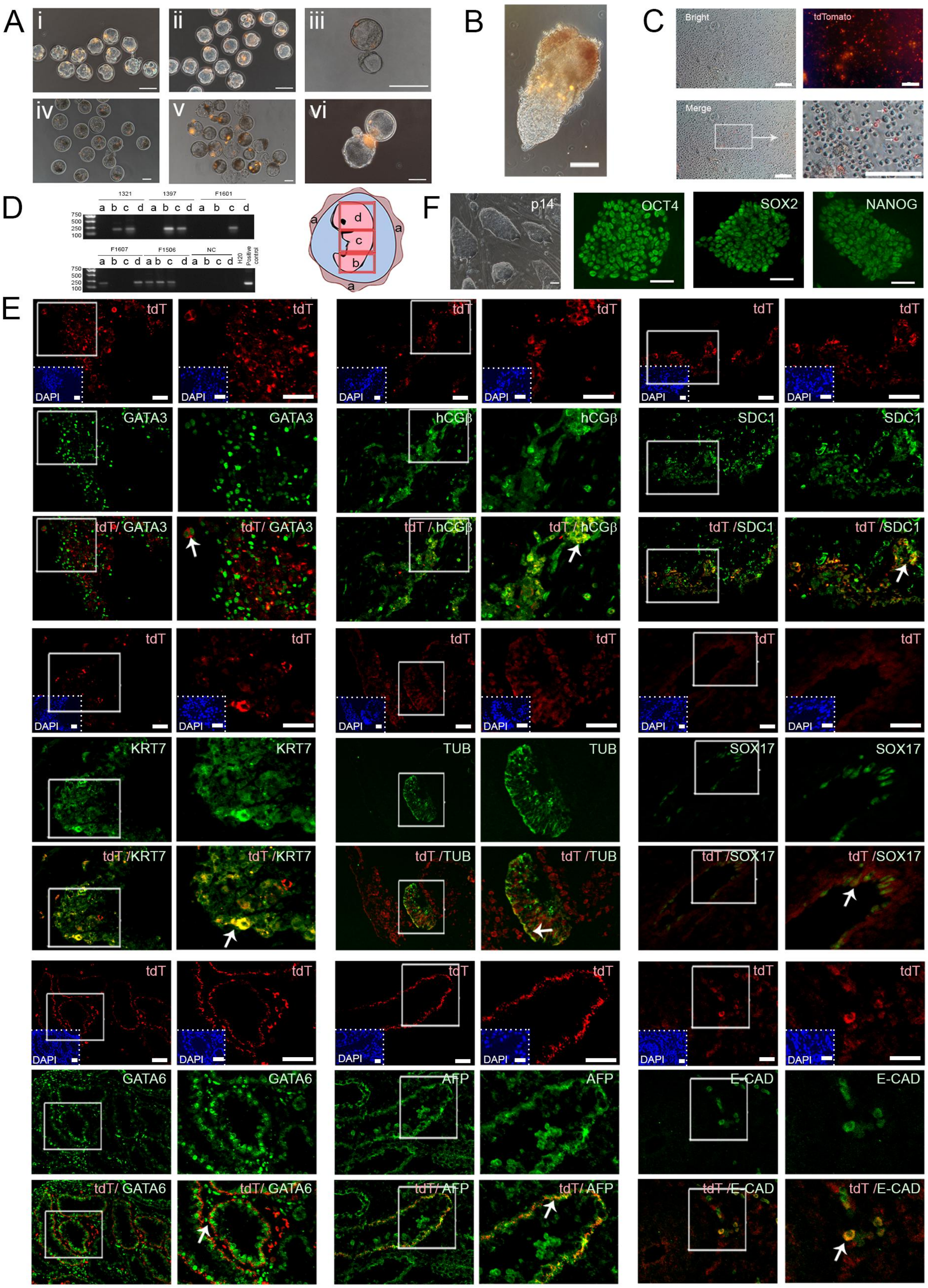

A
i
ii
iii
iv
v
vi
B
C
Bright
tdTomato
Merge
D
1321
1397
F1601
F1607
F1506
NC
a b c d
a
b
c
d
F
p14
OCT4
SOX2
NANOG
E
tdT
DAPI
GATA3
tdT/ GATA3
hCGβ
tdT / hCGβ
SDC1
tdT /SDC1
KRT7
tdT /KRT7
TUB
tdT /TUB
SOX17
tdT /SOX17
GATA6
tdT/ GATA6
AFP
tdT/ AFP
E-CAD
tdT /E-CAD

Fig. S4. Developmental potential of EPSCs in chimeras. (*A*) Injection of bEPSCs to mouse and bovine preimplantation embryos. (i, ii, iii) Injection of tdTomato^{+} donor bEPSC$^{iPS\text{-}Q36}$ to mouse 8-cell stage embryo (i); injected 8-cell stage embryo cultured in KSOM + bEPSCM (1:1) medium for 24 hours (ii); and for 48 hours (iii). (iv, v, vi) Injection of tdTomato^{+} donor bEPSCs$^{ES\text{-}A15}$ to bovine early blastocysts for *in vitro* development. Injected bovine embryos (iv), were cultured in SOF + bEPSCM (1:1) medium for 24 hours to the blastocysts (v, vi). Scale bars, 100 µm. (*B*) A mouse E6.5 embryo that has the tdTomato^{+} cells. The donor bEPSC-originated tdTomato^{+} cells are primarily located in the extra-embryonic ectoderm (ExE) region. Scale bar, 50 µm. (*C*) Dissociated single cells from tdTomato^{+} tissues in chimera no. F1506 were examined under fluorescence microscope to detect tdTomato^{+} cells. Scale bars, 100 µm. (*D*) Genomic DNA PCR genotyping to confirm the presence of tdTomato cells. Genomic DNA was extracted from tissues dissected from embryonic and the extraembryonic tissues: (*a*) chorioallantois membranes; (*b*) caudal; (*c*) trunk; and (*d*) head. Chimeric fetuses were no.1321 (day 72), no.1397 (day 38), no. F1607, no. F1601 and no. F1506 (day 40). (*E*) Immunostaining of cryosections,the chorioallantois membranesof day 40 chimera F1607 for GATA3, hCGß, SDC1 and KRT7; Cryosections of chimera 1397 for AFP and SOX17; Cryosections of chimera 1506 for E-CADHERIN, GATA6 and β-TUBULIN III. tdT: tdTomato, TUB: β-TUBULIN III, E-CAD: E-CADHERIN. Scale bars, 50 µm. (*F*) Secondary bEPSCs at passage 14, which were derived from SCNT blastocysts with bEPSCs as the donor. Immunofluorescence analysis confirms expression of OCT4, NANOG and SOX2. Scale bars, 50 µm.

Table S1. DMRs at the bovine genomic imprinting loci.

Cluster Name	ICR or DMR	Corresponding gene	Chromosome	Sequence coordinates	Reference
DLK1-DIO3	IG-DMR	DLK1, PEG11, RTL1, MEG8, MEG8, DIO3	21	65634498- 65721065	(17)
DLK1-DIO3	MEG3 DMR	Meg3	21	65721065- 65729891	(17)
H19-IGF2	H19 ICR	H19	29	49504869- 49508995	(18-20)
IGF2R	DMR2	IGF2R	9	96220935- 96223721	(19)
SNRPN	DMR	SNRPN	21	1936489-1939007	(21)
PEG3	DMR	PEG3	18	64097692-64102100	(22)
MEST	DMR	PEG1	4	94249282- 94251680	(18, 23)
PLAGL1	DMR	PLAGL1	9	81403579- 81405018	(18, 23)
ZIM2	DMR	ZIM2	18	64021500- 64023080	(24)

The bovine (*Bos taurus*) genome assembly is ARS-UCD1.2 (GCF_002263795.1)

Table S2. bEPSC development in post-implantation chimeras

Group	No. cells of injected	No. recipients	No. pregnant recipients	Pregnancy Rate	No. fetus recovered	Chimeric Rate	Gestation time of the Chimera
Control	/	50	30	60%	0	None	/
Test	5～10	35	13	37%	13	38% (5/13)	day 38 (1)<br>day 40 (3)<br>day 72 (1)

Table S3. Antibodies used in this study

Antigen	Company	Cat No.	Dilution
Anti-Oct-3/4	Santa Cruz Biotechnology	sc-5279	1:200
Anti-trimethyl-H3 (Lys27)	Millipore	07-449	1:200
Anti-Sox2	Millipore	AB5603	1:200
Anti-Nanog	Thermo Fisher Scientific	14-5761-80	1:200
Anti-TuJ-1	R and D Systems	MAB1195	1:200
Anti-α-SMA	R and D Systems	MAB1420	1:200
Anti-AFP	R and D Systems	MAB1368	1;200
Anti-E-cadherin	BD Biosciences	610181	1:100
Anti-CDX-2	BioGenex	AM392	1:200
Anti-SOX17	R and D Systems	AF1924	1:200
Anti-PL-1	Santa Cruz Biotechnology	sc-376436	1:50
Anti-KRT7	Santa Cruz Biotechnology	sc-70936	1:50
Anti-RFP	Rockland	600-401-379	1:200

Table S4. PCR Primer sequences

Gene name	Forward (5'-3')	Reverse (5'-3')
GAPDH	ggagcacgagaggaagagtt	cctcagggccttagagatgg
OCT4	ggggaaactgattggaggga	actaggtcccaggcttcttt
NANOG	ttcctccacccctttttagcc	tgtacttcaacaaaccagcca
SOX2	tgctgcctctttaagactaggac	aaatcaggcgaagaataatttgg
SALL4	catttatccgagcccag	ttggccccatgtgtcatgtaa
KLF2	cgtccttctccactttcgct	ccagggtccgggtagtagaa
KLF4	tcaggagacacaaggaaacca	ccccttggcgttttgtaagt
KLF17	caagcggagatccccagttt	ggtgttgcctgagatggtca
TFCP2L1	ctgcgttcattcaggtgcac	ttttcccggtcggtcttctg
ZIC2	ggtgttgcctgagatggtca	ggtgttgcctgagatggtca
FOXD3	ggtgttgcctgagatggtca	ggtgttgcctgagatggtca
PAX6	gtctgtaccaacgataacatacc	gcctcatctgaatcttctcc
FGF5	atctgcagatctacccggatg	ccccgtcttttcagttctgtg
MIXL1	caggttctgggagcaagaag	tggctgctttgtatgtctgc
HAND1	tctctctgcatcctcgtcct	ggtggtgatgatgtgcgtag
MYF5	agacgcctgaagaaggtcaa	tgccatcagagcaacttgag
CD34	ctggccttcacctctgtctc	aaaaatccaccccatcttcc
HNF4A	ttggtcacagtgaagcaagc	gtgaagaagtgaggcgaagg
FOXA1	ctttcaagcgcagctatcct	gctggttctgccggtaatag
AFP	agcttggtggtggatgagac	acgaccgtctccaattgttc
SOX17	cttcatggtgtgggcgaag	taggagataggacagcgggaa
SOX7	caaggacgagaggaaacgtc	cgtggccggtacttgtagtt
NANOS3	cacctccgtctacagctacac	aagacttcctggcacctttgg
CDX2	gtctggagctggagaaggag	ctttcctttgctctgcggtt
GATA3	taacatcgacggtcaaggcaa	gatggacgtcttggagaaggg
TFAP2C	gtccctcctcagctctacatc	aaagtcccgagccaaatgaac
CGA	actgccctgactacattctgc	ggacagccctgcattgtaaac
PGF	ggaacatttcatcggaggtgg	tcatctccacaaagaagggct
EOMES	gcaaacaacacaacaacacccag	aaggtctgtgtcttagagggc
KRT7	tgaacaaggtggagttggagg	cctcagactgcagctctttca
SDC1	cacagtgtggccgtaaacatg	ggcgtgtgtgatgaggtgata
TFCP2C	gtccctcctcagctctacatc	aaagtcccgagccaaatgaac
BLIMP1	acaccattaagcccatccctg	ttggagcggtacaagtccttc
Genotyping tdTomato	cccgtaatgcagaagaagacca	gatggtgtagtcctcgttgtgg
RT-PCR detection of OMSK	ccccgtggttacctcttctt	ccgcatgttagcagacttcc
RT-PCR detection of RL	ctcaaactggctggggatgt	acgggacaaagctcttccag
RT-PCR detection of NL	tatgtgaaccggtgctagcc	tttggcgagaggggaaagac
OCT4-mCherry Knockin	catctgccctggaatgaacatccctg	cttgtacacatagatggaatagctctc

SI References

1. X. Gao *et al.*, Establishment of porcine and human expanded potential stem cells. *Nat Cell Biol* **21**, 687-699 (2019).
2. W. Wang *et al.*, Rapid and efficient reprogramming of somatic cells to induced pluripotent stem cells by retinoic acid receptor gamma and liver receptor homolog 1. *Proc Natl Acad Sci U S A* **108**, 18283-18288 (2011).
3. Y. S. Bogliotti *et al.*, Efficient derivation of stable primed pluripotent embryonic stem cells from bovine blastocysts. *Proc Natl Acad Sci U S A* **115**, 2090-2095 (2018).
4. Y. Takashima *et al.*, Resetting transcription factor control circuitry toward ground-state pluripotency in human. *Cell* **158**, 1254-1269 (2014).
5. T. W. Theunissen *et al.*, Systematic Identification of Culture Conditions for Induction and Maintenance of Naive Human Pluripotency. *Cell Stem Cell* **15**, 524-526 (2014).
6. Q. L. Ying *et al.*, The ground state of embryonic stem cell self-renewal. *Nature* **453**, 519-523 (2008).
7. S. Bao *et al.*, The germ cell determinant Blimp1 is not required for derivation of pluripotent stem cells. *Cell Stem Cell* **11**, 110-117 (2012).
8. S. Zhang *et al.*, Aberrant DNA methylation reprogramming in bovine SCNT preimplantation embryos. *Sci Rep* **6**, 30345 (2016).
9. S. Bao *et al.*, Derivation of hypermethylated pluripotent embryonic stem cells with high potency. *Cell Res* **28**, 22-34 (2018).
10. R. Lister *et al.*, Human DNA methylomes at base resolution show widespread epigenomic differences. *Nature* **462**, 315-322 (2009).
11. A. Molaro *et al.*, Sperm Methylation Profiles Reveal Features of Epigenetic Inheritance and Evolution in Primates. *Cell* **146**, 1028-1040 (2011).
12. C. Trapnell, L. Pachter, S. L. Salzberg, TopHat: discovering splice junctions with RNA-Seq. *Bioinformatics* **25**, 1105-1111 (2009).
13. S. Anders, P. T. Pyl, W. Huber, HTSeq-a Python framework to work with high-throughput sequencing data. *Bioinformatics* **31**, 166-169 (2015).
14. F. Krueger, S. R. Andrews, Bismark: a flexible aligner and methylation caller for Bisulfite-Seq applications. *Bioinformatics* **27**, 1571-1572 (2011).
15. H. Li *et al.*, The Sequence Alignment/Map format and SAMtools. *Bioinformatics* **25**, 2078-2079 (2009).
16. J. T. Leek, W. E. Johnson, H. S. Parker, A. E. Jaffe, J. D. Storey, The sva package for removing batch effects and other unwanted variation in high-throughput experiments. *Bioinformatics* **28**, 882-883 (2012).
17. M. Zhang *et al.*, An imprinted long noncoding RNA located between genes Meg8 and Meg9 in the cattle Dlk1-Dio3 domain. *Genetica* **145**, 1-7 (2017).
18. A. M. O'Doherty *et al.*, DNA methylation dynamics at imprinted genes during bovine pre-implantation embryo development. *BMC Dev Biol* **15**, 13 (2015).
19. L. C. Smith *et al.*, Developmental and epigenetic anomalies in cloned cattle. *Reprod Domest Anim* **47 Suppl 4**, 107-114 (2012).
20. J. Suzuki, Jr. *et al.*, Loss of methylation at H19 DMD is associated with biallelic expression and reduced development in cattle derived by somatic cell nuclear transfer. *Biol Reprod* **84**, 947-956 (2011).
21. J. Suzuki, Jr. *et al.*, In vitro culture and somatic cell nuclear transfer affect imprinting of SNRPN gene in pre- and post-implantation stages of development in cattle. *BMC Dev Biol* **9**, 9 (2009).
22. M. Kaneda *et al.*, Epigenetic analysis of bovine parthenogenetic embryonic fibroblasts. *J Reprod Dev* **63**, 365-375 (2017).
23. A. M. O'Doherty, L. C. O'Shea, T. Fair, Bovine DNA methylation imprints are established in an oocyte size-specific manner, which are coordinated with the expression of the DNMT3 family proteins. *Biol Reprod* **86**, 67 (2012).

24. J. Kim, A. Bergmann, S. Lucas, R. Stone, L. Stubbs, Lineage-specific imprinting and evolution of the zinc-finger gene ZIM2. *Genomics* **84**, 47-58 (2004).
